# Mathematical discoveries from program search with large language models

**DOI:** 10.1038/s41586-023-06924-6

**Published:** 2023-12-14

**Authors:** Bernardino Romera-Paredes, Mohammadamin Barekatain, Alexander Novikov, Matej Balog, M. Pawan Kumar, Emilien Dupont, Francisco J. R. Ruiz, Jordan S. Ellenberg, Pengming Wang, Omar Fawzi, Pushmeet Kohli, Alhussein Fawzi

**Affiliations:** 1 Google DeepMind, London, UK; 2 Department of Mathematics, University of Wisconsin-Madison, Madison, WI USA; 3 Laboratoire de l’Informatique du Parallélisme, University of Lyon (Inria, ENS Lyon, UCBL, LIP), Lyon, France

**Keywords:** Computer science, Pure mathematics

## Abstract

Large language models (LLMs) have demonstrated tremendous capabilities in solving complex tasks, from quantitative reasoning to understanding natural language. However, LLMs sometimes suffer from confabulations (or hallucinations), which can result in them making plausible but incorrect statements^1,2^. This hinders the use of current large models in scientific discovery. Here we introduce FunSearch (short for searching in the function space), an evolutionary procedure based on pairing a pretrained LLM with a systematic evaluator. We demonstrate the effectiveness of this approach to surpass the best-known results in important problems, pushing the boundary of existing LLM-based approaches^3^. Applying FunSearch to a central problem in extremal combinatorics—the cap set problem—we discover new constructions of large cap sets going beyond the best-known ones, both in finite dimensional and asymptotic cases. This shows that it is possible to make discoveries for established open problems using LLMs. We showcase the generality of FunSearch by applying it to an algorithmic problem, online bin packing, finding new heuristics that improve on widely used baselines. In contrast to most computer search approaches, FunSearch searches for programs that describe how to solve a problem, rather than what the solution is. Beyond being an effective and scalable strategy, discovered programs tend to be more interpretable than raw solutions, enabling feedback loops between domain experts and FunSearch, and the deployment of such programs in real-world applications.

## Abstract (web summary)

FunSearch makes discoveries in established open problems using large language models by searching for programs describing how to solve a problem, rather than what the solution is.

## Main

Many problems in mathematical sciences are ‘easy to evaluate’, despite being typically ‘hard to solve’. For example, in computer science, NP-complete optimization problems admit a polynomial-time evaluation procedure (measuring the quality of the solution), despite the widespread belief that no polynomial-time algorithms to solve such problems exist. We focus in this paper on problems admitting an efficient ‘evaluate’ function, which measures the quality of a candidate solution. Prominent examples include the maximum independent set problem and maximum constraint satisfaction problems (such as finding the ground state energy of a Hamiltonian). Our goal is to generate a ‘solve’ program, such that its outputs receive high scores from the ‘evaluate’ function (when executed on inputs of interest), and ultimately improve on the best-known solutions.

Whereas large language models (LLMs) have recently seen notable improvements in their coding capabilities^4–8^, with applications including debugging^9,10^, solving code competitions^11,12^ and improving code performance^13^, synthesizing ‘solve’ programs for open problems requires finding new ideas that are verifiably correct. This is very hard for LLMs, as they tend to confabulate or ultimately fall short of going beyond existing results. To surpass the ‘nominal’ capabilities of LLMs, recent studies^3^ have combined them with evolutionary algorithms^14,15^, leading to important improvements on diverse synthetic problems^16^, searching for neural network architectures^17–19^ and solving puzzles^20^. Our proposed method, FunSearch, pushes the boundary of LLM-guided evolutionary procedures to a new level: the discovery of new scientific results for established open problems and the discovery of new algorithms. Surpassing state-of-the-art results on established open problems provides a clear indication that the discoveries are truly new, as opposed to being retrieved from the LLM’s training data.

FunSearch (short for searching in the function space) combines a pretrained (frozen) LLM, whose goal is to provide creative solutions, with an evaluator, which guards against confabulations and incorrect ideas. FunSearch iterates over these two components, evolving initial low-scoring programs into high-scoring ones discovering new knowledge. Key to the success of this simple procedure is a combination of several essential ingredients. First, we sample best performing programs and feed them back into prompts for the LLM to improve on; we refer to this as best-shot prompting. Second, we start with a program in the form of a skeleton (containing boilerplate code and potentially known structure about the problem), and only evolve the part governing the critical program logic. For example, by setting a greedy program skeleton, we evolve a priority function used to make decisions at every step. Third, we maintain a large pool of diverse programs by using an island-based evolutionary method that encourages exploration and avoids local optima. Finally, leveraging the highly parallel nature of FunSearch, we scale it asynchronously, considerably broadening the scope of this approach to find new results, while keeping the overall cost of experiments low.

We show the surprising effectiveness of FunSearch on several use cases. We consider a fundamental problem in extremal combinatorics, namely, the cap set problem^21,22^. FunSearch demonstrates the existence of hitherto unknown constructions that go beyond existing ones, including the largest improvement in 20 years to the asymptotic lower bound. This demonstrates that it is possible to make a scientific discovery—a new piece of verifiable knowledge about a notorious scientific problem—using an LLM. Using FunSearch, we also find new algorithms for the online bin packing problem that improve on traditional ones on well-studied distributions of interest^23,24^, with potential applications to improving job scheduling algorithms.

Whereas most computer search techniques output directly what the solution is (for example, a list of vectors forming a cap set), FunSearch produces programs generating the solution. For structured problems, such programs tend to be more interpretable—facilitating interactions with domain experts—and concise—making it possible to scale to large instances—compared to a mere enumeration of the solution. In addition, decision procedures (such as for bin packing) described by code in a standard programming language are crucially easier to deploy compared to other types of descriptions (for example, neural networks), which typically require specialized hardware and for which verifying design specifications is notoriously hard.

## FunSearch

**Fig. 1:**
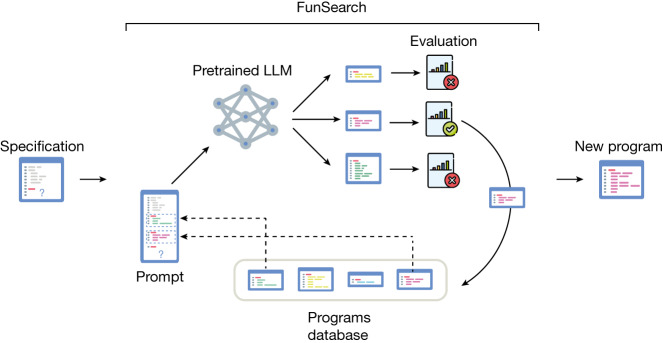
Overview of FunSearch. The input to FunSearch is a specification of the problem in the form of an ‘evaluate’ function, an initial implementation of the function to evolve, which can be trivial, and potentially a skeleton. At each iteration, FunSearch builds a prompt by combining several programs sampled from the programs database (favouring high-scoring ones). The prompt is then fed to the pretrained LLM and new programs are created. Newly created programs are then scored and stored in the programs database (if correct), thus closing the loop. The user can at any point retrieve the highest-scoring programs discovered so far.

An overview of FunSearch is shown in Fig. 1, and its components are described in more detail below. For more details and ablations showing the importance of each component, see Methods and Supplementary Information Appendix A.

### Specification

**Fig. 2:**
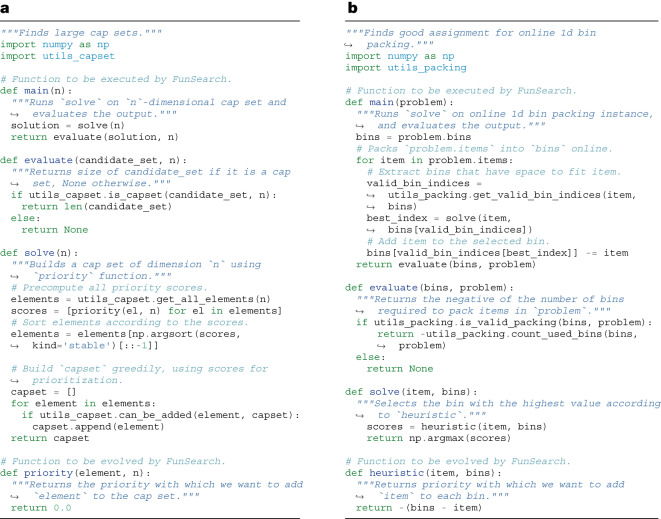
Examples of FunSearch specifications for two problems. The ‘evaluate’ function takes as input a candidate solution to the problem, and returns a score assessing it. The ‘solve’ function contains the algorithm skeleton, which calls the function to evolve that contains the crucial logic. **a**, Cap set. The function to evolve is called ‘priority’. **b**, Online bin packing. The function to evolve is called ‘heuristic’. The ‘main’ function implements the evaluation procedure by connecting the pieces together. Specifically, it uses the ‘solve’ function to solve the problem and then scores the resulting solutions using the ‘evaluate’ function. In the simplest cases, ‘main’ just executes ‘solve’ once and uses ‘evaluate’ to score the output, for example, **a**. In specific settings such as online algorithms, the ‘main’ function implements some more logic, for example, **b**.

The input to FunSearch is a specification of the problem in the form of an ‘evaluate’ function, which scores candidate solutions. In addition, we provide an initial program (which can be trivial) to evolve. Although in principle these are the minimum requirements, we found that performance tends to improve significantly if we write the initial ‘solve’ program in the form of a skeleton (containing boilerplate code and previous knowledge of the problem in the form of a program structure), and only use FunSearch to evolve the critical part that governs its logic. Fig. 2a shows an example in which the skeleton takes the form of a simple greedy algorithm, and the crucial part to evolve by FunSearch is the priority function that is used to make the greedy decision at every step. This delegates to FunSearch precisely the part that is usually the hardest to come up with. Whereas a fixed skeleton may constrain the space of programs that can be discovered, we find it improves overall results because it focuses the LLM resources on only evolving the critical part, instead of also using the LLM to recreate already known program structures (with more opportunities for mistakes that would render the entire program incorrect). If available, the user can optionally provide extra known information about the problem at hand, in the form of docstrings, relevant primitive functions or import packages, which FunSearch may use.

### Pretrained LLM

The LLM is the creative core of FunSearch, in charge of coming up with improvements to the functions presented in the prompt and sending these for evaluation. We obtain our results with a pretrained model, that is, without any fine-tuning on our problems. We use Codey, an LLM built on top of the PaLM2 model family^25^, which has been fine-tuned on a large corpus of code and is publicly accessible through its API^26^. Because FunSearch relies on sampling from an LLM extensively, an important performance-defining tradeoff is between the quality of the samples and the inference speed of the LLM. In practice, we have chosen to work with a fast-inference model (rather than slower-inference, higher-quality), and the results in the paper are obtained using a total number of samples on the order of 10^6^. Beyond this tradeoff, we have empirically observed that the results obtained in this paper are not too sensitive to the exact choice of LLM, as long as it has been trained on a large enough corpus of code. See Supplementary Information Appendix A for a comparison to StarCoder^6^, a state-of-the-art open-source LLM for code.

### Evaluation

Programs generated by the LLM are evaluated and scored on a set of inputs. For example, in the cap set problem (‘Extremal combinatorics’ section) the inputs are the values of the dimensionality *n* that we are interested in, and in combinatorial optimization (‘Bin packing’ section), the inputs correspond to different bin packing instances. The scores across different inputs are then combined into an overall score of the program using an aggregation function, such as the mean. The scored programs are then sent to the programs database. Programs that were incorrect (that did not execute within the imposed time and memory limits, or produced invalid outputs) are discarded, and the remaining scored programs are then sent to the programs database.

### Programs database

The programs database keeps a population of correct programs, which are then sampled to create prompts. Preserving and encouraging diversity of programs in the database is crucial to enable exploration and avoid being stuck in local optima. To encourage diversity, we adopt an islands model, also known as a multiple population and multiple-deme model^27,28^, which is a genetic algorithm approach. Several islands, or subpopulations, are created and evolved independently. To sample from the program database, we first sample an island and then sample a program within that island, favouring higher-scoring and shorter programs (see Methods for the exact mechanism). Crucially, we let information flow between the islands by periodically discarding the programs in the worst half of the islands (corresponding to the ones whose best individuals have the lowest scores). We replace the programs in those islands with a new population, initialized by cloning one of the best individuals from the surviving islands.

### Prompt

New prompts are created by ‘best-shot prompting’ from the programs database, and are then fed to the LLM to generate a new program. We first sample *k* programs from a single island in the programs database, according to the procedure described above. Sampled programs are then sorted according to their score, and a version is assigned to each (‘v0’ for the lowest scoring program, ‘v1’ for the second lowest scoring and so on). These programs are then combined into a single prompt—with the version appended as a suffix to the function name; for example, in the case of Fig. 2a, this would be ‘priority_v0’, ‘priority_v1’, ...—and the header of the function we wish to generate (for example, ‘priority_vk’) is added to the end of the prompt. In practice, we set *k* = 2, as two functions lead to better results compared to just one, with diminishing returns beyond that. Constructing a prompt by combining several programs (as opposed to only one) enables the LLM to spot patterns across the different programs and generalize those. Related approaches to prompt building have been recently considered, for example ref. ^16^, and were shown to perform well on different domains.

### Distributed approach

We implement FunSearch as a distributed system that has three types of workers—a programs database, samplers and evaluators—which communicate asynchronously. The programs database stores and serves programs, samplers generate new functions using the pretrained LLM and evaluators assess programs, as shown in Supplementary Fig. F.26. In the example shown in Fig. 2a, the programs database stores priority functions, samplers generate new implementations of ‘priority’ and evaluators score the proposals by executing the ‘main’ function on user-specified inputs. Our distributed system offers several advantages. First, it naturally leverages parallelism across different tasks: for example, LLM sampling and evaluation are performed concurrently. Second, it enables scaling to more than one sampler and evaluator, which would be a very limiting setup, considering that evaluation can take minutes for many problems of interest. Running evaluators in parallel considerably broadens the scope of this approach to such problems. The distributed setting enables the running of many evaluator nodes on inexpensive CPU hardware, whereas few samplers run on machines with accelerators for fast LLM inference; this keeps the overall cost and energy usage of experiments low. In our experiments, we typically use 15 samplers and 150 CPU evaluators (can be served on five CPU servers each running 32 evaluators in parallel). See Supplementary Information Appendix A for more details. Also, because of the randomness of LLM sampling and the evolutionary procedure, for some problems we run several experiments to get the best reported results. See Methods and Supplementary Information Appendix A.3 for a full statistical analysis.

We now describe some of the new discoveries made by FunSearch in two different fields: pure mathematics and applied computer science. Further discoveries on other problems (namely, the corners problem and Shannon capacity of cycle graphs) are presented in Supplementary Information Appendix B. The full discovered programs are available in Supplementary Information Appendix C.

## Extremal combinatorics

We apply FunSearch to two related problems in extremal combinatorics: a branch of mathematics that studies the maximal (or minimal) possible sizes of sets satisfying certain properties.

### Cap sets

**Fig. 3:**
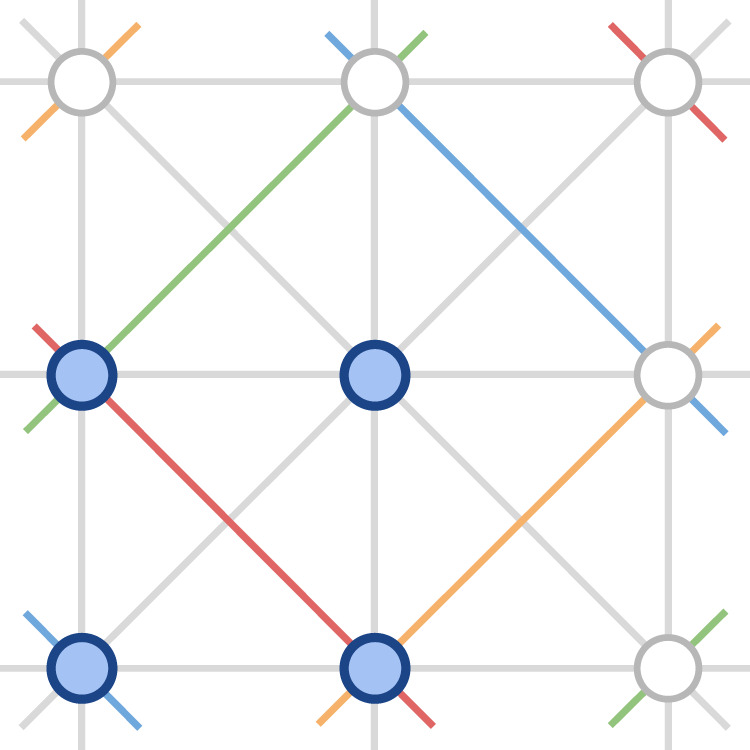
Diagram of a cap set of size four in ${{\mathbb{Z}}}_{3}^{2}$. The circles are the elements of ${{\mathbb{Z}}}_{3}^{2}$ with the ones belonging to the cap set shown in blue. The possible lines in ${{\mathbb{Z}}}_{3}^{2}$ are also shown (with colours indicating lines that wrap around in arithmetic modulo 3). No three elements of the cap set are in a line.

The cap set problem^21^, once described by Terence Tao as ‘perhaps my favourite open question’^29^, refers to the task of finding the largest possible set of vectors in ${{\mathbb{Z}}}_{3}^{n}$ (known as a cap set) such that no three vectors sum to zero. Geometrically, no three points of a cap set are in a line (see Fig. 3 for an example with *n* = 2).

The problem has drawn much interest for a variety of reasons. For one, it is an analogue of the classical number theory problem of finding large subsets of primes in which no three are in arithmetic progression. For another, it differs from many problems in combinatorics in that there is no consensus among mathematicians about what the right answer should be. Finally, the problem serves as a model for the many other problems involving ‘three-way interactions’. For instance, progress towards improved upper bounds for the cap set problem^30,31^ immediately led to a series of other combinatorial results, for example, on the Erdös–Radio sunflower problem^32^.

The exact size of the largest possible cap set in *n* dimensions is known only for *n* ≤ 6. A brute force approach is not practical as the search space quickly becomes enormous with growing *n*, for example, around 3^1,600^ for *n* = 8. Previous methods impose potentially suboptimal restrictions on the search space^33,34^. By contrast, we search the full space by means of an algorithm skeleton that uses a function ‘priority’ : ${{\mathbb{Z}}}_{3}^{n}\to {\mathbb{R}}$. Intuitively, this function provides a priority with which each $x\in {{\mathbb{Z}}}_{3}^{n}$ should be included in the cap set. Our algorithm starts with an empty set and iteratively adds the vector $x\in {{\mathbb{Z}}}_{3}^{n}$ with the highest priority that does not violate the cap set constraint; Fig. 2a. Starting from a trivial constant function, we evolve the crucial ‘priority’ component of our approach to result in large cap sets.

**Fig. 4:**
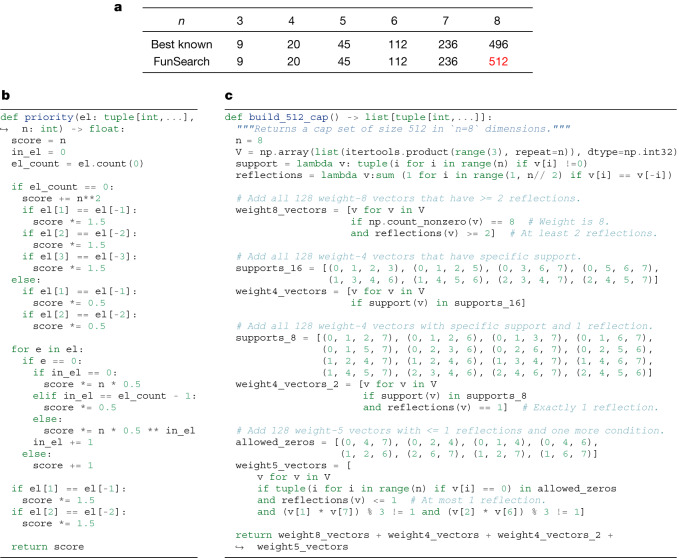
Result of applying FunSearch to the cap set problem. **a**, Size of the largest cap set in ${{\mathbb{Z}}}_{3}^{n}$ for different dimensions *n*. **b**, The function ‘priority’ : ${{\mathbb{Z}}}_{3}^{n}\to {\mathbb{R}}$ discovered by FunSearch that results in a cap set of size 512 in *n* = 8 dimensions. One feature to note is that the priority is affected by whether the same entry appears in positions i and −i (−i denotes the *i*th position counting from the end). This motivates the notion of reflections, used in **c**. **c**, An explicit construction of this new 512-cap, which we were able to manually construct thanks to having discovered the cap set by searching in function space. See Supplementary Information Appendix E.2 for more details and for relation to Hill cap.

Using this approach, we discovered cap sets of sizes shown in Fig. 4a. Notably, in dimension *n* = 8, FunSearch found a larger cap set than what was previously known, thus illustrating the power of FunSearch to discover new constructions. This also shows the scalability of FunSearch to larger dimensions, in which the previously best-known construction relied on a complex combination of cap sets in lower dimensions^33,34^. By contrast, FunSearch discovered a larger cap set from scratch, without having to be explicitly taught any way of combining cap sets. Moreover, we do not just discover the set of 512 eight-dimensional vectors in itself, but a program that generates it: we show this program in Fig. 4b. Through inspecting the code, we obtain a degree of understanding of what this set is: specifically, manual simplification of Fig. 4b provides the construction in Fig. 4c. Some properties of this construction are similar to the construction of the Hill cap^35,36^, which results in the optimal 112-cap in ${{\mathbb{Z}}}_{3}^{6}$.

### Admissible sets

Beyond finding the size of the largest cap set *c*_*n*_ in dimension *n*, a fundamental problem in additive combinatorics^22^ is determining the capacity $C=\mathop{\sup }\limits_{n}\,{c}_{n}^{1/n}$. The breakthrough result from ref. ^31^ established an upper bound of *C* ≤ 2.756. In this work, we are interested in lower bounds on *C*. To this end, we use the framework of constant weight admissible sets (or admissible sets for short)^34,37^, which has established the current state-of-the-art.

Formally, admissible sets ${\mathcal{A}}(n,w)$ are collections of vectors in {0, 1, 2}^*n*^ satisfying two properties: (1) each vector has the same number *w* of non-zero elements but a unique support (therefore $$| A| \le \left(\begin{array}{c}n\\ w\end{array}\right)$$); (2) for any three distinct vectors there is a coordinate in which their three respective values are {0, 1, 2}, {0, 0, 1} or {0, 0, 2}. Informally, an admissible set describes how to combine cap sets in smaller dimensions into large cap sets in higher dimensions^34^. We denote the set of full-size admissible sets (with $$| A| =\left(\begin{array}{c}n\\ w\end{array}\right)$$) as ${\mathcal{I}}(n,w)$. The current state-of-the-art^38^ has relied on SAT solvers to construct large admissible sets.

**Fig. 5:**
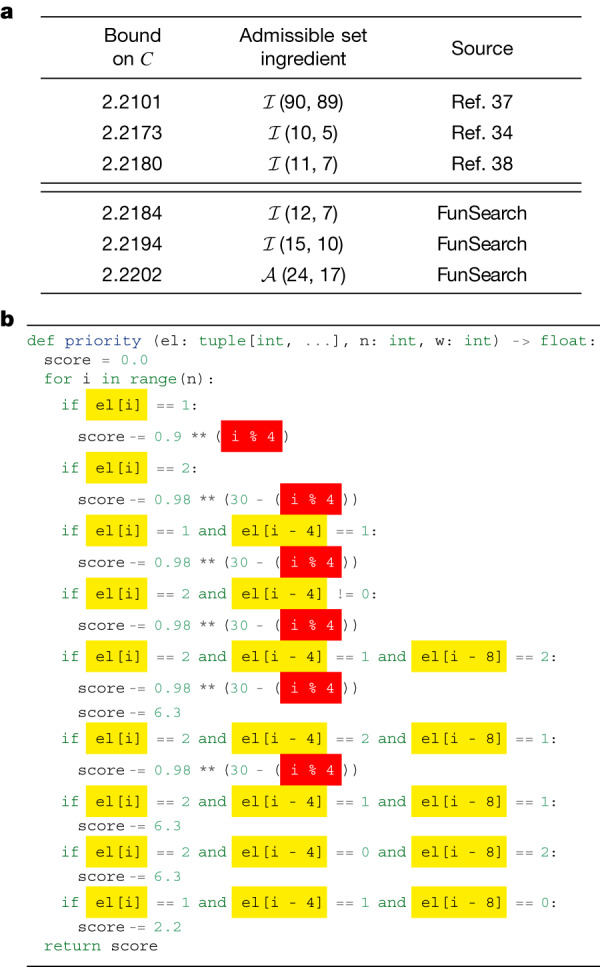
Results on the cap set problem through admissible sets. **a**, Summary of lower bounds on the cap set capacity *C*. **b**, The ‘priority’ function ${\{0,1,2\}}^{n}\to {\mathbb{R}}$ discovered by FunSearch that results in an ${\mathcal{I}}(12,7)$ admissible set. The source code shows that when *n* = 12, the function treats the four triples of coordinates {0, 4, 8}, {1, 5, 9}, {2, 6, 10} and {3, 7, 11} together. We then checked that the admissible set is in fact symmetric under independent cyclic permutations of coordinates within each of these four triples. See Supplementary Information Appendices D and  E.3 for more details.

As before, we evolve a function ‘priority’ : ${\{0,1,2\}}^{n}\to {\mathbb{R}}$, which is used to iteratively grow admissible sets. Starting from a trivial constant function, we discover one that provides us with an ${\mathcal{I}}(12,7)$ admissible set; the discovered program is shown in Fig. 5b. This discovery alone already improves the lower bound on the cap set capacity from 2.2180 (ref. ^38^) to 2.2184. Yet, interpreting the program found by FunSearch (Fig. 5b) helps us significantly push the boundaries of what admissible sets we can construct. Specifically, we notice that the discovered ‘priority’ function treats the *n* coordinates in a highly symmetric way, and indeed it turns out that the admissible set it constructs is preserved under independent cyclic permutations of coordinates within four disjoint groups of coordinate triples. Hereinafter we call such admissible sets symmetric (see Supplementary Information Appendix D for a formal definition).

We now use FunSearch to directly search for symmetric admissible sets. Note that this is a more restricted and also much smaller search space, which allows for significantly higher dimensions and weights than were previously possible. This led us to discovering a full-size ${\mathcal{I}}(15,10)$ admissible set (indicating *C* ≥ 2.219486) and a partial admissible set in ${\mathcal{A}}(24,17)$ of size 237,984, which implies a new lower bound on the cap set capacity of 2.2202 (Fig. 5a). Although this is a great improvement to the lower bound compared to research in the last 20 years, we note it is still far from the upper bound and we hope our results inspire future work on this problem.

Not only does FunSearch scale to much larger instances than traditional combinatorial solvers (Supplementary Information Appendix A.4), but it is also a unique feature of searching in function space that we were able to inspect the code discovered by FunSearch and infer a new insight into the problem, in the form of a new symmetry. The procedure we followed in this section is a concrete example of how LLM-based approaches can be used in mathematical sciences: FunSearch suggests a solution, which is examined by researchers, who may note features of interest. These features are used to refine the search, leading to better solutions. This process can be iterated, with both human and search consistently in the loop.

## Bin packing

Combinatorial optimization is a subfield of mathematics that plays an important role across a wide range of areas, from theoretical computer science to practical problems in logistics and scheduling. Whereas many combinatorial optimization problems are provably hard to solve for large instances, it is typically possible to achieve strong performance using heuristics to guide the search algorithm. The choice of a heuristic is crucial for obtaining strong performance, but designing a good heuristic is difficult in practice. In this section, we show that FunSearch can be used to discover effective heuristics for one of the central problems in combinatorial optimization: bin packing^39^.

The goal of bin packing is to pack a set of items of various sizes into the smallest number of fixed-sized bins. Bin packing finds applications in many areas, from cutting materials to scheduling jobs on compute clusters. We focus on the online setting in which we pack an item as soon as it is received (as opposed to the offline setting in which we have access to all items in advance). Solving online bin packing problems then requires designing a heuristic for deciding which bin to assign an incoming item to.

Heuristics for online bin packing are well studied and several variants exist with strong worst case performance^40–45^. However, they often show poor performance in practice^39^. Instead, the most commonly used heuristics for bin packing are first fit and best fit. First fit places the incoming item in the first bin with enough available space, whereas best fit places the item in the bin with least available space where the item still fits. Here, we show that FunSearch discovers better heuristics than first fit and best fit on simulated data.

To achieve this, we define a heuristic as a program that takes as input an item and an array of bins (containing the remaining capacity of each bin) and returns a priority score for each bin. The ‘solve’ function picks the bin with the highest score according to the heuristic (Fig. 2b). FunSearch is then used to evolve this heuristic, starting from best fit.

We first evaluate FunSearch on the well-known OR-Library bin packing benchmarks^23^, consisting of four datasets, OR1 to OR4, containing bin packing instances with an increasing number of items (see Supplementary Information Appendix E.4 for details). We evolve our heuristic on a training set of generated bin packing instances with the same number of items as those in OR1 and, after the evolutionary process is concluded, test it on the OR1 to OR4 datasets. We measure performance as the fraction of excess bins used over the *L*_2_ lower bound^46^ of the optimal offline packing solution (which is generally not achievable in the online setting).

As can be seen in Table 1, FunSearch outperforms both first fit and best fit across all datasets. Further, the learned heuristic generalizes: even though it has only seen instances of the same size as OR1 during training, it generalizes across problem sizes, performing even better on large instances and widening the gap to best fit. In addition to the OR benchmarks, we also use FunSearch to evolve heuristics on bin packing instances sampled from a Weibull distribution, as these closely follow many real-world scheduling problems^24,47^ (see Supplementary Information Appendix E.4 for details). As shown in Table 1, the performance of FunSearch is very strong on this dataset, significantly outperforming first fit and best fit across instances, as well as scaling gracefully to large instances (being only 0.03% off the lower bound on the optimum for 100,000 items). In addition, FunSearch is robust and consistently outperforms these baselines as shown in the statistical analysis in the Supplementary Information Appendix A.3.

**Table 1:** Online bin packing results

	OR1	OR2	OR3	OR4	Weibull 5k	Weibull 10k	Weibull 100k
First fit	6.42%	6.45%	5.74%	5.23%	4.23%	4.20%	4.00%
Best fit	5.81%	6.06%	5.37%	4.94%	3.98%	3.90%	3.79%
**FunSearch**	**5.30%**	**4.19%**	**3.11%**	**2.47%**	**0.68%**	**0.32%**	**0.03%**

Fraction of excess bins (lower is better) for various bin packing heuristics on the OR and Weibull datasets. FunSearch outperforms first fit and best fit across problems and instance sizes.

**Fig. 6:**
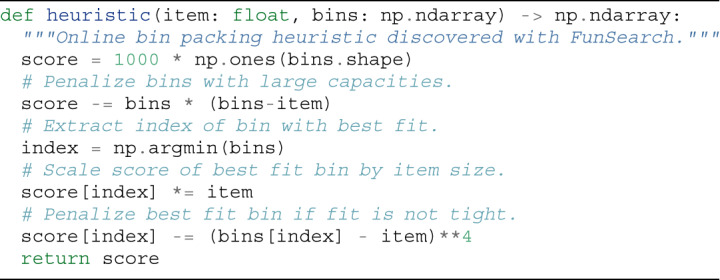
Example of a short online bin packing heuristic discovered by FunSearch for the OR dataset. This example illustrates frequently observed behaviour: instead of always packing items into the best fit bin, the heuristic encourages packing the item only if the fit is tight. Comments in the code were manually added. See Supplementary Information Appendix C for more discovered heuristics.

We observed that several heuristics discovered by FunSearch use the same general strategy for bin packing (see Fig. 6 for an example). Instead of packing items into bins with the least capacity (such as best fit), the FunSearch heuristics assign items to least capacity bins only if the fit is very tight after placing the item. Otherwise, the item is typically placed in another bin, which would leave more space after the item is placed. This strategy avoids leaving small gaps in bins that are unlikely to ever be filled (see Supplementary Information Appendix E.5 for example visualizations of such packings).

As this example demonstrates, the benefits of FunSearch extend beyond theoretical and mathematical results to practical problems such as bin packing. Indeed, bin packing, and related combinatorial optimization problems, are ubiquitous and find applications across a range of industries. We are optimistic that FunSearch could be applied to several such use cases with potential for real-world impact.

## Discussion

The effectiveness of FunSearch in discovering new knowledge for hard problems might seem intriguing. We believe that the LLM used within FunSearch does not use much context about the problem; the LLM should instead be seen as a source of diverse (syntactically correct) programs with occasionally interesting ideas. When further constrained to operate on the crucial part of the algorithm with a program skeleton, the LLM provides suggestions that marginally improve over existing ones in the population, which ultimately results in discovering new knowledge on open problems when combined with the evolutionary algorithm. Another crucial component of the effectiveness of FunSearch is that it operates in the space of programs: rather than directly searching for constructions (which is typically an enormous list of numbers), FunSearch searches for programs generating those constructions. Because most problems we care about are structured (highly non-random), we believe that solutions are described more concisely with a computer program, compared to other representations. For example, the trivial representation of the admissible set ${\mathcal{A}}(24,17)$ consists of more than 200,000 vectors, but the program generating this set consists of only a few lines of code. Because FunSearch implicitly encourages concise programs, it scales to much larger instances compared to traditional search approaches in structured problems. In a loose sense, FunSearch attempts to find solutions that have low Kolmogorov complexity^48–50^ (which is the length of the shortest computer program that produces a given object as output), whereas traditional search procedures have a very different inductive bias. We believe that such Kolmogorov-compressed inductive bias is key to FunSearch scaling up to the large instances in our use cases. In addition to scale, we have empirically observed that FunSearch outputs programs that tend to be interpretable: that is, they are clearly easier to read and understand compared to a list of numbers. For example, by scrutinizing FunSearch’s output for the admissible set problem, we found a new symmetry, which was then subsequently used to improve the results even further. Despite the rarity of symmetric solutions, we observe that FunSearch preferred symmetric ones, as these are more parsimonious (that is, they require less information to specify), in addition to the natural bias of LLMs (trained on human-produced code) in outputting code with similar traits to human code. This is in contrast to traditional genetic programming that does not have this bias (and in addition requires hand-tuning the mutation operators^51^).

We note that FunSearch, at present, works best for problems having the following characteristics: (1) availability of an efficient evaluator; (2) a ‘rich’ scoring feedback quantifying the improvements (as opposed to a binary signal) and (3) ability to provide a skeleton with an isolated part to be evolved. For example, the problem of generating proofs for theorems^52–54^ falls outside this scope, because it is unclear how to provide a rich enough scoring signal. By contrast, for MAX-SAT, the number of satisfied clauses can be used as a scoring signal. In this paper, we have explicitly striven for simplicity and we are confident that FunSearch can be further extended to improve its performance and be applicable to more classes of problems. In addition, the rapid development of LLMs is likely to result in samples of far superior quality at a fraction of the cost, making FunSearch more effective at tackling a broad range of problems. As a result, we foresee that automatically tailored algorithms will soon become common practice and deployed in real-world applications.

## Methods

### Implementation details of FunSearch

#### Distributed system

We implement FunSearch as a distributed system that has three types of workers: a programs database, samplers and evaluators. The programs database stores the initial user-provided program, as well as all programs received from the evaluators. The samplers are in charge of performing the LLM inference step; to do so they repeatedly query the programs database for prompts. To achieve higher sampling throughput, samplers generate several samples from each prompt. The samples from the LLM (that is, the generated programs) are sent to the evaluators, which score programs by executing them on inputs of interest and assessing the outputs using ‘evaluate’. Programs that are correct are sent to the programs database to be stored. Each of the three FunSearch components is provided as both Python code and pseudocode (Supplementary Information Appendix F).

#### Prompt building

When queried for a prompt, the programs database samples *k* programs to encourage the LLM to merge ideas from them (we typically set *k* = 2; Supplementary Information Appendix E.1). Programs are sorted according to their score in increasing order, starting from version 0 (‘v0’). Using these *k* programs, the prompt is built as explained next.

**Extended Data Fig. 1:**
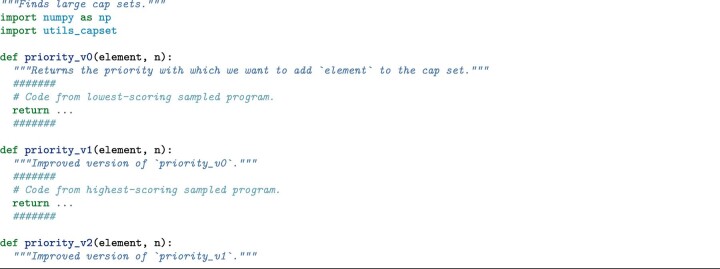
Example of best-shot prompting, based on the skeleton from Fig. 2a. The prompt includes *k* = 2 implementations sampled from the programs database, with higher-scoring implementations being more likely to be included.

For the sake of clarity, we use here the problem specification from Fig. 2a to precisely describe the prompting mechanism. The overall structure of the prompt mimics the structure of the program skeleton, with the following differences: (1) the ‘priority’ function is stripped out and replaced with the *k* = 2 programs sampled, first ‘priority_v0’ and then ‘priority_v1’. (2) After that, a ‘priority_v2’ function with no body is appended: the LLM will be in charge of completing the body of that function. (3) All other functions that appear before ‘priority_v0’ are removed. See Extended Data Fig. 1 for an example of the structure of a prompt.

#### Evolutionary method and program selection

**Extended Data Fig. 2:**
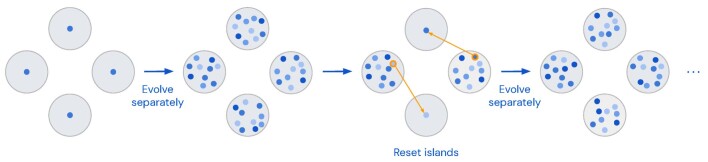
Evolutionary method. The initial programs are separated into islands and each of them is evolved separately. After a number of iterations, the islands with the worst score are wiped and the best program from the islands with the best score are placed in the empty islands. Evolution then proceeds separately again until the next reset. This process is repeated until termination.

Another key feature of FunSearch is the method used for evolution of the population of programs from the programs database, as well as for program selection: that is, how the programs database samples programs when queried for a prompt. For this, we use the islands model, a parallel genetic algorithm^27,28^. Specifically, we split the population into *m* separate groups or islands. Each island is initialized with a copy of the user-provided initial program and is evolved separately. That is, whenever a prompt is required, we first uniformly sample an island and then sample *k* = 2 programs from that island to build the prompt. The programs generated from the LLM on the basis of that prompt will later be stored in the same island. Every 4 h, we discard all the programs from the *m*/2 islands whose best instances have the lowest score. Each of these islands is then seeded with a single program, obtained by first choosing one of the surviving *m*/2 islands uniformly at random and then retrieving the highest-scoring program from that island (breaking ties in favour of older programs). The evolutionary process is then restarted from this state, in which the reset islands contain one high-performing program each (Extended Data Fig. 2).

This method has several advantages. First, drawing the analogy in which an island corresponds to an experiment, this approach effectively allows us to run several smaller experiments in parallel instead of a single large experiment. This is beneficial because single experiments can get stuck in local minima, in which most programs in the population are not easily mutated and combined into stronger programs. The multiple island approach allows us to bypass this and effectively kill off such experiments to make space for new ones starting from more promising programs. Second, promising experiments are run for longer, as the islands that survive a reset are the ones with higher scores.

**Extended Data Fig. 3:**
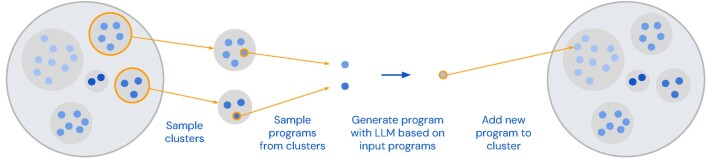
Program clusters within islands. Within each island, programs are grouped into clusters based on their signature (i.e., their scores on several inputs). We first sample clusters, favoring the ones with higher score. Within the chosen clusters, we sample a program, favoring shorter programs. The sampled programs are used to prompt the LLM which generates a new program. If the new program is correct, it is added to the island, either in an existing cluster or a new one if its signature was not yet present.

Within each island, we further cluster programs according to their signature. We define the signature of a program as the tuple containing the program’s scores on each of the inputs (for example, the cap set size for each input *n*). Programs with the same signature are clustered together. When sampling a program within an island, we first sample an island’s cluster and then a program within that cluster (Extended Data Fig. 3). This approach, which aims to preserve diversity^55,56^, is related to Lexicase^57^ in that both approaches consider a set of test cases for scoring an individual, and it is related to fitness uniform optimization^58^, which also clusters individuals on the basis of their fitness value; however, we sample the clusters on the basis of their score instead of uniformly, as detailed next.

When sampling a cluster, we favour those with larger score values. Specifically, let *s*_*i*_ denote the score of the *i*th cluster, defined as an aggregation (for example, mean) of all the scores in the signature that characterizes that cluster. The probability *P*_*i*_ of choosing cluster *i* is

1 $${P}_{i}=\frac{\exp ({s}_{i}/{T}_{{\rm{c}}{\rm{l}}{\rm{u}}{\rm{s}}{\rm{t}}{\rm{e}}{\rm{r}}})}{{\sum }_{{i}^{{\prime} }}\exp ({s}_{{i}^{{\prime} }}/{T}_{{\rm{c}}{\rm{l}}{\rm{u}}{\rm{s}}{\rm{t}}{\rm{e}}{\rm{r}}})},{T}_{{\rm{c}}{\rm{l}}{\rm{u}}{\rm{s}}{\rm{t}}{\rm{e}}{\rm{r}}}={T}_{0}\left(1-\frac{n\,\,{\rm{m}}{\rm{o}}{\rm{d}}\,\,N}{N}\right),$$

where *T*_cluster_ is the temperature parameter, *n* is the current number of programs in the island, and *T*_0_ and *N* are hyperparameters (given in Supplementary Information Appendix E.1). This approach is sometimes referred to as the Boltzmann selection procedure^59^.

When sampling a program within a cluster, we favour shorter programs. In particular, let *ℓ*_*i*_ denote the negative length of the *i*th program within the chosen cluster (measured as the number of characters), and let ${\widetilde{{\ell }}}_{i}=\frac{{{\ell }}_{i}-\mathop{\min }\limits_{{i}^{{\prime} }}{{\ell }}_{{i}^{{\prime} }}}{\mathop{\max }\limits_{{i}^{{\prime} }}{{\ell }}_{{i}^{{\prime} }}+1{0}^{-6}}$. We set the probability of each program proportional to $\exp ({\widetilde{{\ell }}}_{i}/{T}_{{\rm{program}}})$, where *T*_program_ is a temperature hyperparameter.

#### Robustness

Owing to randomness in LLM sampling and in the evolutionary procedure, repeating an experiment can lead to different results. For some problems (for example, cap set through the admissible set problem and online bin packing) every single run of FunSearch surpasses the baseline, with only some variation in the magnitude of the difference. For example, all experiments on admissible sets improve on the previous best capacity lower bound, with 60% of experiments on ${\mathcal{I}}(12,7)$ finding a full-size admissible set. For other problems, many independent repetitions of an experiment may be necessary to improve on previous best results. In particular, the case of cap set by direct construction in *n* = 8 dimensions is particularly challenging, with only four out of 140 experiments discovering a cap set of size 512. See Supplementary Information Appendix A.3 for more details.

### Related work

#### LLMs

The rise of powerful LLMs such as that in ref. ^60^ has been followed by systems in which an LLM core has been enveloped by a ‘programmatic scaffold’^61^, and several LLM calls were connected in some way to accomplish larger and more intricate tasks beyond what would be possible using a single prompt and the raw LLM, possibly by using external tools or external memory streams^62–66^. LLMs have also been paired with evaluators; for example, refs. ^20,67^ fine-tuned an LLM on data that had been previously generated by the LLM itself (respectively on puzzle problems and solutions, and on justifications and/or explanations for answers to questions), and they used an evaluator to assess the correctness of this data, ensuring that the fine-tuning dataset contained only correct solutions and/or explanations. More related to our approach is the use of LLMs as mutation operators on code, and ref. ^3^ was the first study to show that coupling an LLM with a programmatic way of scoring a solution can lead to a self-improvement loop. In refs. ^16–19^, the LLM was used as a crossover operator rather than a mutation one, that is, the LLM prompts are composed of several functions, similarly to FunSearch. In refs. ^3,16^, the task was to improve code that generated bidimensional virtual robots that could move as far as possible in a given simulated terrain (ref. ^16^ also considered the tasks of symbolic regression, natural language sentences and image generation). In refs. ^17–19^ the task was to find neural network architectures (described with Python code), and in ref. ^68^ the task was continuous exploration in the game of Minecraft. By contrast, in this paper, we tackle open problems in mathematics and algorithm design, and we surpass human-designed constructions. We achieve that by combining several ingredients: a distributed system with many samplers and evaluators that communicate asynchronously, a user-provided program specification and skeleton, as well as an evolutionary mechanism based on islands that preserves the diversity of programs. FunSearch achieves that using an off-the-shelf LLM without fine-tuning.

More broadly, LLMs have been used for program synthesis as one of its main applications^4–8^. There are many use cases being explored, such as automatically editing code to improve performance^13^, automatically debugging code^9,10^, generating code from natural language descriptions^69–71^ and doing so to solve problems in code competitions^11,12^. Unlike the above approaches that provide tools to increase the productivity of software engineers, we combine in this paper the creativity of LLMs with the power of evolutionary procedures to push the boundaries of human knowledge through solving open hard problems. Another line of research uses LLMs to guide the search for formal proofs for automatic theorem proving^52–54^. Although this approach has the potential to eventually find new knowledge, the achievements of these methods still lag behind the frontier of human knowledge.

#### Genetic programming

Genetic programming is a subfield of computer science concerned with automatically generating or discovering computer programs using evolutionary methods^15,72,73^ and is used for symbolic regression applications^74,75^ and discovery of optimization algorithms^76^ among others. In this broad sense, combining LLMs with evolution can be seen as an instance of genetic programming with the LLM acting as a mutation and crossover operator. However, using an LLM mitigates several issues in traditional genetic programming^51^, as shown in Supplementary Information Appendix A and discussed in ref. ^3^. Indeed, genetic programming methods require defining several parameters, chief among them the set of allowed mutation operations (or primitives)^15^. Designing such a set of operations is non-trivial and problem specific, requiring domain knowledge about the problem at hand or its plausible solution^51^. Although research has been done to mitigate this limitation, through, for example, the reuse of subprograms^77^ or modelling the distribution of high-performing programs^78^, designing effective and general code mutation operators remains difficult. By contrast, LLMs have been trained on vast amounts of code and as such have learned about common patterns and routines from human-designed code. The LLM can leverage this, as well as the context given in the prompt, to generate more effective suggestions than the random ones typically used in genetic programming.

Related to genetic programming, the field of hyper-heuristics^79,80^ seeks to design learning methods for generating heuristics applied to combinatorial optimization problems. In practice, these heuristics are often programs discovered through genetic programming, typically by evolving a heuristic on a set of instances of a given combinatorial optimization problem, such as bin packing^81^. Indeed, like FunSearch, hyper-heuristics have also been applied to online bin packing, with the learned heuristics able to match the performance of first fit^82^ and best fit^83^ on a set of generated bin packing instances. Augmenting the heuristics with memory of previously seen items can even lead to heuristics outperforming best fit^84^. In addition, these evolved heuristics can sometimes generalize to larger instances than the ones they were trained on^85^, similar to the learned FunSearch heuristics. However, as is the case with genetic programming, one of the fundamental limitations of hyper-heuristics is that the components of the evolved heuristic must be manually defined by the user and often need to be tailored to a specific problem to be effective. The LLM in FunSearch allows us to bypass this limitation and learn heuristics for bin packing and job scheduling as well as discovering new mathematical constructions, all within a single pipeline without problem-specific tuning.

#### Program superoptimization and software engineering

Searching for the best way of modifying source code is a task that appears in several branches of computer science and software development. These occurrences can be broadly classified into two groups: first, in which the goal is to find semantic-preserving modifications (this arises in program optimization and superoptimization, in which the aim is to modify the program so that it executes faster while maintaining its input–output behaviour), and second, in which the goal is to find programs with different semantics (this arises, for example, in automatic program repair and mutation testing). With some exceptions discussed below, most of these areas use relatively simple and hard-coded mutation operators on either the source code directly (such as deleting or swapping lines) or on the abstract syntax tree.

Machine learning approaches have been used for program superoptimization. For example, ref. ^86^ used reinforcement learning to learn the sampling probabilities used within a hierarchical probabilistic model of simple program edits introduced by STOKE^87^. Neural networks have also been proposed as a mutation operator for program optimization in ref. ^88^. These studies operated on code written in Assembly (perhaps because designing meaningful and rich edit distributions on programs in higher-level languages is challenging). More recently, ref. ^13^ used LLMs to find performance-improving edits to code written in C++ or Python. We also note that reinforcement learning has recently been applied to discover new faster algorithms for fundamental operations such as matrix multiplication^89^ and sorting^90^.

In this paper, we have not explicitly explored semantic-preserving applications such as discovering performance-improving code edits, but we believe that FunSearch could be an effective method for that setting too. In both use cases presented in the main text, the goal is to evolve programs with new semantics, but the application is different from program repair or mutation testing: in the ‘Extremal combinatorics’ section, we used FunSearch to discover a program that constructs a previously unknown mathematical object, and in the ‘Bin packing’ section, we used FunSearch to discover a program that corresponds to a more efficient heuristic for online bin packing.

## Online content

Any methods, additional references, Nature Portfolio reporting summaries, source data, extended data, supplementary information, acknowledgements, peer review information; details of author contributions and competing interests; and statements of data and code availability are available at 10.1038/s41586-023-06924-6.

### Supplementary information

Supplementary Information Further details about the method and extra results.

Supplementary Data 1 This zipped code file contains: (a) the evolutionary algorithm, code manipulation routines and a single-threaded implementation of the FunSearch pipeline; and (b) output functions of interest produced by FunSearch.

## Data Availability

The experiments carried out in this paper do not require any data corpus other than the publicly available OR-Library bin packing benchmarks^23^. The output functions of interest produced by FunSearch are shown across the main paper and in text files in the Supplementary Information.

## Code availability

The discovered functions as well as the evolutionary algorithm, code manipulation routines and a single-threaded implementation of the FunSearch pipeline are available as Python code in the Supplementary Information and at https://github.com/google-deepmind/funsearch. Furthermore, the software library launchpad^91^ and a sandbox for safely executing generated code on our internal distributed system were used. No training or fine-tuning of a LLM is required; API access for inference is sufficient. We used Codey^26^, which is available through its API, and StarCoder^6^, which is open source.
