# Supplementary material for: Mathematical discoveries from program search with large language models
Source: Nature. 2023 Dec 14;625(7995):468–75. doi: 10.1038/s41586-023-06924-6 (PMC10794145; doi:10.1038/s41586-023-06924-6)
Supplement: Supplementary file 1 — Further details about the method and extra results. [file 41586_2023_6924_MOESM1_ESM.pdf]

nature portfolio

https://doi.org/10.1038/s41586-023-06924-6

# Supplementary information

# Mathematical discoveries from program search with large language models

In the format provided by the authors and unedited

# Mathematical discoveries from program search with large language models (Supplementary material)

## Contents


**A Analysis** **2**
- A.1 Ablations . . . 2
- A.2 Choice of LLM . . . 3
- A.3 Statistical analysis . . . 5
- A.4 Comparison with traditional solvers . . . 6
- A.5 Distributed setup and energy usage . . . 7

**B Other results** **7**
- B.1 Shannon capacity of cycle graphs . . . 7
- B.2 Corners problem . . . 8

**C Skeletons and discovered programs** **9**
- C.1 Cap sets . . . 9
- C.2 Admissible sets . . . 10
- C.3 Combinatorial optimization . . . 14
- C.4 Shannon capacity of cycle graphs . . . 16
- C.5 Corners problem . . . 18

**D Symmetric admissible sets and pre-admissible sets** **20**

**E More details** **23**
- E.1 Hyperparameters . . . 23
- E.2 Explicit construction of a size-512 cap set in $\mathbb{Z}_3^8$ . . . 24
- E.3 Conception of symmetric admissible sets . . . 27
- E.4 Bin packing datasets . . . 28
- E.5 Bin packing visualizations . . . 29

**F Implementation of *FunSearch*** **29**
- F.1 Sampler . . . 30
- F.2 Evaluator . . . 31
- F.3 ProgramsDB . . . 31
  - F.3.1 Island . . . 34
  - F.3.2 Cluster . . . 36

# A Analysis

Here we present an analysis of *FunSearch*, focusing on five aspects:

1. Importance of methodological choices in the design of *FunSearch*, showcased through ablations of individual components (Appendix A.1).
2. Effect of the choice of LLM, together with a comparison to a non-LLM based mutation operator (Appendix A.2).
3. Robustness of *FunSearch*, specifically the frequency with which it discovers the best results on both extremal combinatorics and combinatorial optimization (Appendix A.3).
4. Scalability of *FunSearch*, specifically its ability to solve larger problem instances than traditional approaches. We illustrate this by comparing to the SAT-based approach for finding large admissible sets (Appendix A.4).
5. Distributed nature of *FunSearch*, and the resulting cost and energy usage of running an experiment with *FunSearch* (Appendix A.5).

## A.1 Ablations

We carry out ablations on the task of finding the full-sized symmetric admissible set $\mathcal{I}(15, 10)$. Recall that *FunSearch* consists of the following components, whose efficacy we wish to understand via the means of ablation.

- **Skeleton-based approach.** In order to improve the chances of *FunSearch* to find the full-sized $\mathcal{I}(15, 10)$, we provide a program skeleton that isolates the part of the code that needs to be improved, namely, the `priority` function (see Figure C.10). The `priority` function assigns a priority to include each potential vector in the admissible set. In order to understand the importance of the skeleton-based approach, we use an alternative problem specification where *FunSearch* is required to produce code that directly outputs an ordered list of all the potential vectors. The symmetric admissible set is then constructed in a greedy fashion by considering the vectors in the order provided and including the current one if it does not violate any constraints with respect to the previously added vectors. We refer to this approach as *W/O Skeleton.*
- **Evolutionary approach.** *FunSearch* keeps a population of correctly generated programs in the programs database, and then combines them in a prompt to obtain new programs. In order to understand the importance of evolving the prompts, we consider an alternative method where only the initial (user-provided) prompt is used to generate a large number of programs, which are then analyzed to identify the best one. We refer to this approach as *W/O Evolution.*
- **Maintaining population diversity.** *FunSearch* adopts an approach based on an islands model, where the islands with the lowest scoring programs are periodically killed and reinitialized using the higher scoring ones. This encourages diversity among the programs generated as each island evolves programs independently, while also allowing them to communicate with each other periodically. In order to understand the importance of maintaining population diversity, we consider an alternative method that has only a single island. We refer to this approach as *Less Diversity* (as some diversity is still maintained through clustering within the island).

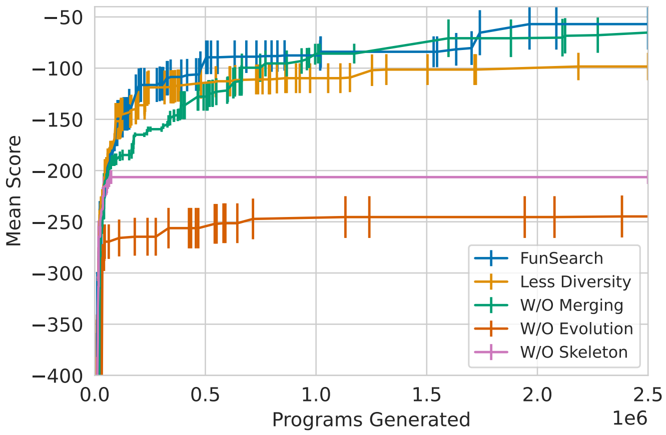


**Figure A.1:** Ablation study of *FunSearch* for finding the full-sized admissible set $\mathcal{I}(15, 10)$. The $x$-axis shows the number of programs generated, while the $y$-axis shows the difference between the size of the largest admissible set computed thus far, and the size of $\mathcal{I}(15, 10)$ (equal to $\binom{15}{10}$), averaged over 5 random seeds (higher is better), as well as the corresponding standard error bars. The curve 'FunSearch' refers to the overall *FunSearch* approach, while the other curves correspond to its ablated variants (see text for details). As can be seen, the use of a skeleton and the evolution of prompts are particularly critical for finding a good solution. With less diversity in the prompts, we are able to obtain large but not a full-sized admissible set in any of the five runs.

- **Prompt building.** When constructing a new prompt, *FunSearch* samples $k = 2$ programs from the programs database in order to allow the LLM to merge information from multiple programs when creating a new one. To understand the importance of using multiple programs in a single prompt, we consider an alternative approach where only a single program is used to construct the prompt (i.e., $k = 1$). We refer to this approach as *W/O Merging*.

None of the variants were able to discover a full-sized $\mathcal{I}(15, 10)$ admissible set, with the exception of the *W/O Merging* variant, which found it in 1 out of 5 runs. In comparison, *FunSearch* found it in 2 out of 5 runs, and achieved a higher average size of the largest admissible set found than other variants (see Figure A.1). We also note that in other tasks, such as computing cap sets in dimensions $n = 8$, the *W/O Merging* variant was less successful at obtaining the best results compared to *FunSearch*.

## A.2 Choice of LLM

A key part of *FunSearch* is the use of an LLM to generate programs. This raises two important questions. First, is the choice of the LLM critical to its performance? Second, is an LLM even needed?

In order to answer the first question, we compare the performance of two LLMs: Codey [1], which is the default model used in our experiments, and StarCoder [2], which is an open-sourced model with 15 billion parameters available for free to all researchers.

In order to answer the second question, we compare the performance of *FunSearch* (which uses LLMs to propose program improvements), with an alternative approach that does not use LLMs, but instead relies on evolving programs via a hand-designed distribution of random mutations. To

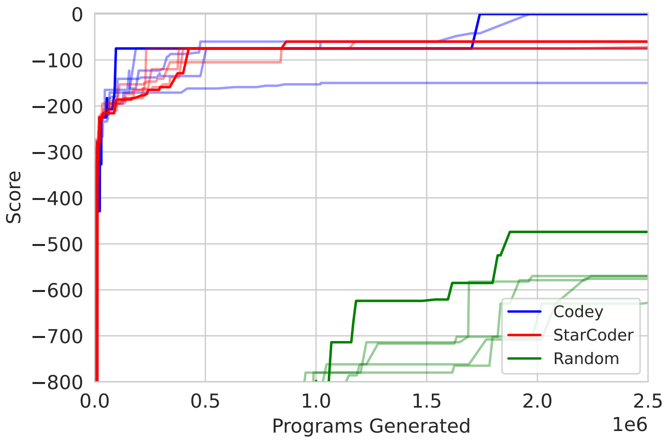


**Figure A.2:** Ablation study of *FunSearch* for finding the full-sized admissible set $\mathcal{I}(15, 10)$. The $x$-axis shows the number of programs generated, while the $y$-axis shows the difference in the size of the largest admissible set found so far and $\mathcal{I}(15, 10)$ (higher is better). Five independent runs are shown for each model, with the best run amongst each five (i.e., the one that provides the largest admissible set the fastest) highlighted as a solid non-translucent line. Note that two of the five runs for 'Codey' result in a full-sized admissible set. While 'StarCoder' is not able to find the full-sized admissible set in any of the five runs, it still finds large admissible sets that improve upon the previous state of the art lower bound on the cap set capacity. This illustrates that *FunSearch* is robust to the choice of the model as long as it has been trained sufficiently well to generate code.

this end, we use mutations similar to [3]. In particular, we define a list of possible binary operations (addition, multiplication, division, integer division, exponentiation, and modulo) and a list of unary operations (indexing of arrays and several common NumPy functions [4]: `np.log(x)`, `np.sum(x)`, `np.argmin(x)`, `np.argmax(x)`, `np.median(x)`, `np.exp(x)`, `np.cumsum(x)`, `np.max(x)`, `np.min(x)`, `np.abs(x)`, `np.sign(x)`, `np.negative(x)`, `np.square(x)`, and `np.sqrt(x)`) that can be used. To produce a single mutation we parse the input program with the AST Python parser [5] and insert a new variable at a location chosen at random, assigning to it the result of a new operation constructed as follows. We pick a unary or a binary operation uniformly at random, and for each of its arguments we choose either an already existing variable or a constant (which we draw from either the $\mathcal{N}(0, 1)$ normal distribution, or uniformly from $\{-10, \ldots, 10\}$). Additionally, for every existing operation we delete it with probability 0.1, and for every existing binary operation we change it to another one from the list with probability 0.2, or change one of its arguments (to a randomly chosen existing variable or a random constant) with probability 0.2. We tuned this mutation process trying to make it possible to reproduce some of the best programs discovered by *FunSearch*, but even with reference solutions in mind we were always discovering additional corner cases or unsupported operations which we would have to manually add, which emphasizes how much problem-specific effort the LLM is saving.

Figure A.2 shows the results of the two LLMs and random program mutations, in five different runs of each on the $\mathcal{I}(15, 10)$ problem. Note that StarCoder performs slightly worse compared to Codey: in particular, unlike Codey, the StarCoder-based *FunSearch* is not able to find the full-sized admissible set in any of the runs. However, it is still able to find very large admissible sets that yield an improvement over the previous state-of-the-art cap set capacity lower bound. The

use of an LLM is however clearly critical, as evidenced by the worse performance of the random mutations approach.[1] That said, even though random mutations were clearly inferior to LLMs in results and required a lot more manual tweaking to make them work, their results still exceeded our expectations, indicating the power of other ingredients of *FunSearch* (e.g., the skeleton structure and the evolution pipeline).

## A.3 Statistical analysis

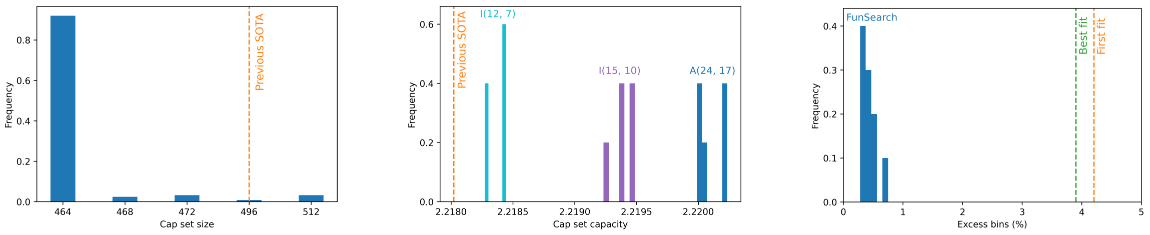


**(a)** Cap set in 8 dimensions. Higher is better.

**(b)** Admissible sets $(n, w)$ of various dimensions $n$ and weights $w$. Higher is better.

**(c)** Online bin packing for the Weibull distribution. Lower is better.

**Figure A.3:** Results across multiple runs of *FunSearch*. (a) Histogram of the size of the cap set of dimension $n = 8$ found across 140 experiments. The task is highly challenging, as demonstrated by the fact that only 4 out of the 140 experiments yield the new state of the art result of size 512. (b) Histogram of the lower bound of the capacity for the cap set problem, computed using various admissible sets. The light blue histogram corresponds to 5 runs for computing $\mathcal{I}(12, 7)$ without imposing symmetry. Note that 3 out of the 5 runs find the complete admissible set. The purple histogram corresponds to 5 runs for computing the symmetric $\mathcal{I}(15, 10)$. In this case, 2 out of the 5 runs find the complete admissible set. Finally, the dark blue histogram corresponds to 5 runs for computing the partial $\mathcal{A}(24, 17)$, which establishes the new state of the art for the lower bound of the capacity. (c) Histogram of the excess bins percentage across 10 runs of the online bin packing problem. In all 10 runs, *FunSearch* finds a better heuristic than the two standard ones used in the literature, namely, first fit and best fit.

The use of an LLM as well as our strategy of sampling the prompts from the database makes the overall *FunSearch* approach stochastic in nature. It is therefore natural to ask how frequently it can provide us with the state of the art results. To answer this, we run *FunSearch* multiple times on the following problems.

- Finding large cap sets in $n = 8$ dimensions. Recall that *FunSearch* obtains a cap set of size 512, thereby establishing a new state of the art for this task.
- Computing the full admissible set $\mathcal{I}(12, 7)$ without explicitly imposing symmetry (which were in fact discovered by analyzing the $\mathcal{I}(12, 7)$ obtained), the full symmetric admissible set $\mathcal{I}(15, 10)$, and the partial admissible set $\mathcal{A}(24, 17)$, which provides the new state of the art lower bound on the cap set capacity.

[1]Even after leaving "Random" generate more than 50 million programs, none of the runs were able to surpass −240 and reached a plateau.

- Generating a heuristic for online bin packing for the Weibull datasets. In the main paper, we demonstrated that *FunSearch* can surpass long established baselines such as first fit and best fit on this task.

Figure A.3 shows the results obtained by *FunSearch* across the multiple runs. As can be seen, the problem of computing large cap sets appears to be particularly challenging, with less than 1/30 experiments providing a new state of the art cap set of size 512 of dimensionality 8. The difficulty of this problem is also reflected in the fact that the lower bound of capacity of the cap set problem has been typically established indirectly by computing large admissible sets instead. Indeed, even for *FunSearch*, the problem of finding large admissible sets turns out to be significantly easier. In particular, all 15 runs across the various dimensionalities and weights provide a better lower bound than the previous state of the art. It is worth noting that for the problem of finding $\mathcal{I}(12, 7)$ without imposing symmetry, all 3 runs that provide the full admissible set do so by computing a symmetric one. This is not due to a large majority of admissible sets being symmetric by nature. In fact, it is quite the opposite with less than 1% of full $\mathcal{I}(12, 7)$ sets satisfying the symmetry property. This can be seen by the fact that, given an admissible set such that the $k$-th coordinate of its $i$-th and $j$-th elements are 1 and 2 respectively, one can swap these coordinate values while still maintaining admissibility. The fact that *FunSearch* consistently finds these rare admissible sets shows its propensity to exploit problem symmetry where available. Finally, *FunSearch* is also highly effective for finding accurate heuristics for online bin packing. Across the 10 runs shown in Figure A.3c, the excess bins percentage obtained using the heuristic found by *FunSearch* is 0.44%±0.11% when evaluated on the Weibull 10k dataset[2]. In comparison, the excess bins percentage for first fit and best first are 4.20% and 3.90% respectively. These results confirm that *FunSearch* is able to discover the reported new results repeatedly.

## A.4 Comparison with traditional solvers

As mentioned in the main paper, one of the advantages of *FunSearch* is its scalability, that is, its ability to solve problems with enormous search spaces. In order to illustrate this advantage, we compare *FunSearch* with a traditional solver for finding large admissible sets. As noted by [6], this can be achieved by formulating the problem as a SAT instance, which enables the use of off-the-shelf solvers. In our comparison, we use the state of the art CP-SAT solver [7], which is publicly available.

We tested two different versions of SAT formulation for admissible sets: (i) the natural encoding of the problem as a SAT instance without any additional constraints, and (ii) an encoding with additional, restrictive constraints handcrafted in [6] "by studying smaller examples of admissible sets, heuristic arguments and a healthy dose of educated guesswork". (There are three such sets of constraints in [6], and we report the best results across the three.) For every admissible set size and SAT formulation we picked 10 random (binary) variables and ran $2^{10}$ different instances of CP-SAT in parallel, each fixing the variables in one of the $2^{10}$ possible ways, and running the solvers for at least 50 hours each.

Table A.4 shows the results. As can be seen, while the SAT solver can be used successfully to address small to medium sized problems, it is unable to handle larger problems. The reason for the success of *FunSearch* is two-fold: (i) searching in the program space instead of attempting to compute the optimal value of a large number of variables in a mathematical optimization program; and (ii) the interpretability of the resulting programs, which enables the further restriction of the search to more concise spaces such as symmetric admissible sets.

[2]Note that we train on datasets with 5k items and test on datasets with 10k items. As such our heuristic must generalize across instance sizes and we therefore exclude functions that explicitly use the number of bins in the heuristic.

| Method | Method variant | Admissible set $\mathcal{I}(n, w)$ (9, 5) | (10, 6) | (11, 7) | (12, 7) | (15, 10) |
|---|---|---|---|---|---|---|
| Parallelized SAT | out of the box | ✓ | × | × | × | × |
| *FunSearch* | out of the box | ✓ | ✓ | ✓ | ✓ | × |
| Parallelized SAT | w/ handcrafted constraints of [6] | ✓ | ✓ | ✓ | × | × |
| *FunSearch* | w/ *FunSearch*-discovered symmetry | ✓ | ✓ | ✓ | ✓ | ✓ |

**Figure A.4:** Comparison of a parallelized SAT solver and *FunSearch* on the problem of finding admissible sets $\mathcal{I}(n, w)$. In both regimes – with and without additional constraints – *FunSearch* scales to significantly larger instances.

## A.5 Distributed setup and energy usage

Finally, we note the importance of employing a distributed approach within *FunSearch*. Finding the full-sized symmetric admissible set $\mathcal{I}(15, 10)$ required the generation and analysis of approximately two million programs. Without parallelization of both the samplers and the evaluators, it would not have been possible to realize this result. It is worth noting, however, that parallelization does not make *FunSearch* prohibitively expensive. To reproduce admissible set experiments done above (generating 2 million samples) one would have to use 15 instances of StarCoder-15B running on A100 40 GB GPU each and 5 CPU servers (each running 32 evaluators in parallel) for two days. We estimate that when running on Google Cloud, the price of an experiment is around \$800 – \$1400, and the energy usage around 250 – 500 kWh; i.e., 0.5% of the energy used for training StarCoder [2]. With further engineering, we believe this cost and energy usage can be reduced significantly. Moreover, in the upcoming years, advancements in LLMs as well as new inference hardware will lower the cost even further.

# B Other results

## B.1 Shannon capacity of cycle graphs

The problems studied in the "Extremal combinatorics" section of the main paper are tightly related to the problem of finding the Shannon capacity of a graph. The Shannon capacity of a graph is an important quantity from Information Theory as it indicates the amount of information that can be transmitted over a noisy channel with zero probability of error [8–11]. Consider a discrete noisy communication channel in which certain symbols can be confused with each other, with a graph describing these confusion patterns. The vertices of the graph correspond to symbols (inputs of the channel), and the edges indicate which symbols can be confused with each other at the receiver side of the communication channel. Formally, the Shannon capacity of a graph $\mathcal{G}$ is defined as $\Theta(\mathcal{G}) = \sup_n \sqrt[n]{\alpha(\mathcal{G}^{\boxtimes n})}$, where $\mathcal{G}^{\boxtimes n}$ denotes the $n$-th strong product of the graph, and $\alpha(\mathcal{G}^{\boxtimes n})$ denotes its independence number (i.e., the size of the largest independent set).

One of the most studied graphs with unknown Shannon capacity is the cycle $\mathcal{C}_m$, i.e., the graph with $m$ nodes and $m$ edges consisting of a single cycle. Determining the Shannon capacity of cycle graphs is a notorious open problem in extremal combinatorics [12, 13]. For even values of $m$, the Shannon capacity of $\mathcal{C}_m$ is $m/2$; however for odd values of $m$, only the capacity for the cycle graph of 5 nodes is known: Shannon showed the lower bound $\Theta(\mathcal{C}_5) \geq \sqrt{5}$ in 1956 [8] and Lóvasz proved the upper bound $\Theta(\mathcal{C}_5) \leq \sqrt{5}$ in 1979 [14], therefore concluding $\Theta(\mathcal{C}_5) = \sqrt{5}$. The Shannon capacity

of the cycle graph $\mathcal{C}_m$ for odd values of $m \geq 7$ remains unknown, although numerous bounds have been proved [12, 14–20].

We apply *FunSearch* to find a function `priority` : $\{0, 1, \ldots, m-1\}^n \to \mathbb{R}$, with the aim of constructing large independent set in powers of cycle graphs $\mathcal{C}_m^{\boxtimes n}$, thereby finding lower bounds on the Shannon capacity of cycle graphs. We discover the following results:

- For the cycle graph of 7 nodes, we find a program that outputs an independent set of size 367 for $\mathcal{C}_7^{\boxtimes 5}$, therefore recovering the best known lower bound $\Theta(\mathcal{C}_7) \geq \sqrt[5]{367}$ [20]. Unlike the method from [20], which finds a large independent set on a related circular graph and then uses some heuristics to project it down to an independent set in $\mathcal{C}_7^{\boxtimes 5}$, the program discovered by *FunSearch* is simpler as it operates directly in $\mathcal{C}_7^{\boxtimes 5}$, thus opening the door for easier interpretability of this construction.
- For the cycle graph of 9 nodes, we find *a single program* that outputs independent sets achieving the best known lower bounds of $\alpha(\mathcal{C}_9^{\boxtimes n})$ for $n = 3, \ldots, 7$, i.e., $\alpha(\mathcal{C}_9^{\boxtimes 3}) \geq 81$, $\alpha(\mathcal{C}_9^{\boxtimes 4}) \geq 324$, $\alpha(\mathcal{C}_9^{\boxtimes 5}) \geq 1458$, $\alpha(\mathcal{C}_9^{\boxtimes 6}) \geq 6561$, and $\alpha(\mathcal{C}_9^{\boxtimes 7}) \geq 26244$. (These bounds are taken from [19] and extrapolated for $n = 6$ and $n = 7$ by taking products of smaller powers of the graph.) The program discovered by *FunSearch* is surprisingly simple —just a couple of lines of code— and provides the state-of-the-art results for multiple powers of the graph. Again, we believe this provides an opportunity for mathematicians for further analysis, understanding, and possibly improvement of the solutions.
- For the cycle graph of 11 nodes, we find a program that provides an independent set of size 754 on $\mathcal{C}_{11}^{\boxtimes 4}$, therefore improving upon the best existing lower bound, $\alpha(\mathcal{C}_{11}^{\boxtimes 4}) \geq 748$ [16, 19].

The programs achieving these results are listed in Appendix C.

## B.2 Corners problem

The corners problem is a related problem in extremal combinatorics, with connections to communication complexity [21–28]. The problem is to find the largest corner-free set in $\mathbb{Z}_p^n \times \mathbb{Z}_p^n$,[3] where a corner is a triple of elements $(x, y)$, $(x + \lambda, y)$, $(x, y + \lambda)$ (in arithmetic modulo $p$), with $x, y, \lambda \in \mathbb{Z}_p^n$ and $\lambda \neq 0$. As for many other problems in mathematics and extremal combinatorics such as the cap set problem, the main quantity of interest is the capacity — the asymptotic rate of growth of the largest corner-free set in $\mathbb{Z}_p^n \times \mathbb{Z}_p^n$ as $n \to \infty$.

The best known asymptotic lower bounds for the corners problem on $\mathbb{Z}_2^n \times \mathbb{Z}_2^n$ and $\mathbb{Z}_3^n \times \mathbb{Z}_3^n$ were obtained using the method of combinatorial degenerations [27]. The combinatorial degenerations method attempts to find the largest set of vertices in a specific hypergraph (i.e., a generalization of a graph in which edges can connect to more than two vertices) describing the problem, such that vertices in the set satisfy certain feasibility constraints [27, Section 2.3]. Any set $S$ of vertices satisfying those constraints implies a lower bound on the capacity of the corners problem, $C \geq \sqrt[n]{|S|}$, which in turn means that the size of the largest corner-free set in $\mathbb{Z}_p^n \times \mathbb{Z}_p^n$ grows asymptotically at least as $C^n/\text{poly}(n)$ when $n \to \infty$.

We use *FunSearch* to guide a greedy search approach to find the largest possible set of vertices in the hypergraph that satisfy the combinatorial degeneration constraints. Similarly to the previous sections, we attempt to find a function `priority` : $\mathbb{Z}_p^n \times \mathbb{Z}_p^n \to \mathbb{R}$ that assigns a priority score to each candidate vertex of the hypergraph. The greedy approach starts with an empty list of indices (each representing a vertex), and at each iteration it adds the valid index (i.e., guaranteeing the combinatorial degeneration constraints) with the highest priority, until it is not possible to add more. The priority heuristic found by *FunSearch* directly leads to new lower bounds on the capacity for the corners problem; see Table B.1. In particular, we find $C \geq \sqrt[4]{137} \approx 3.421$ for $\mathbb{Z}_2$ and

[3] The corners problem can be defined for any abelian group, but here we restrict our attention to $\mathbb{Z}_p$.

| $n$ | Best known [27] size | capacity | *FunSearch* size | capacity |
|---|---|---|---|---|
| 1 | 3 | 3 | 3 | 3 |
| 2 | 11 | 3.317 | 11 | 3.317 |
| 3 | 39 | 3.391 | 39 | 3.391 |
| 4 | – | – | **137** | **3.421** |

**(a)** Results in $\mathbb{Z}_2$.

| $n$ | Best known [27] size | capacity | *FunSearch* size | capacity |
|---|---|---|---|---|
| 1 | 7 | 7 | 7 | 7 |
| 2 | – | – | **53** | **7.280** |
| 3 | – | – | **370** | **7.179** |

**(b)** Results in $\mathbb{Z}_3$.

**Table B.1:** *FunSearch* improves the best known capacity for the corners problem. The table shows the largest set of vertices satisfying the combinatorial degenerations constraints for multiple powers $n$ of the corners hypergraph, as well as the implied lower bounds on the capacity.

$C \geq \sqrt{53} \approx 7.280$ for $\mathbb{Z}_3$, which improve upon the previously best lower bounds of 3.391 and 7, respectively.

# C Skeletons and discovered programs

This section shows functions discovered by *FunSearch* for different problems described in the paper. Note that we have lightly edited these functions by removing unused lines, renaming some variables, and adding comments, in order to increase their readability.

Throughout this section, the decorators `@funsearch.run` and `@funsearch.evolve` are just a way to indicate the main entry point of the program and the function that *FunSearch* should evolve, respectively.

## C.1 Cap sets

In Figure C.6 below we show the full version of the program skeleton in Figure 2 (a). One point to note is that in case the `priority` function leads to ties between elements of $\mathbb{Z}_3^n$, these ties are broken by using the lexicographical order of the elements. Another point to note is that it is perhaps surprising that we can obtain good cap set constructions using priority functions that do not explicitly take into account which specific elements have already been added to the cap set. When choosing the next element of $\mathbb{Z}_3^n$ to add, the current state is taken into account *only* to give a yes or no answer as to whether the element next in line is allowable. Searching this simple class of programs yields surprisingly good results, and at least for now we were not able to do any better by allowing programs that take the current state into account in a more sophisticated way.

```
def priority(el: tuple[int, ...], n: int) -> float:
  """Returns the priority with which we want to add `el` to the cap set."""
  el = np.array(el, dtype=np.float32)
  weight = (el @ el) % 3  # Weight (mod 3) of the full vector.
  a = n // 3
  b = n - n // 3
  s_1 = (el[:b] @ el[:b]) % 3  # Weight (mod 3) of first two thirds.
  s_3 = (2 * (el[:a] @ el[:a])) % 3  # Double norm of first third.
  s_4 = (el[:a] @ el[a:b]) % 3  # Cross correlation.
  s_5 = np.sum(el[:a] == el[-1]) % 3
  return - 3 ** 3 * s_1 + 3 ** 2 * weight + 3 ** 3 * s_3 + 3 ** 2 * s_4 + s_5
```

**Figure C.5:** Priority function that yields a cap set of size 1082 in $n = 9$ dimensions. For each `el` $\in \mathbb{Z}_3^n$ it returns the priority with which we should include `el` in the cap set. Similarly to the priority function yielding a 512-cap in $\mathbb{Z}_3^8$ shown in Figure 4 (a), this priority function also partitions the coordinates into multiple groups, in this case three groups of size three each.

```python
"""Finds large cap sets."""
import itertools
import numpy as np


@funsearch.run
def evaluate(n: int) -> int:
  """Returns the size of an `n`-dimensional cap set."""
  capset = solve(n)
  return len(capset)


def solve(n: int) -> np.ndarray:
  """Returns a large cap set in `n` dimensions."""
  all_vectors = np.array(list(itertools.product((0, 1, 2), repeat=n)), dtype=np.int32)

  # Powers in decreasing order for compatibility with `itertools.product`, so
  # that the relationship `i = all_vectors[i] @ powers` holds for all `i`.
  powers = np.array([3 ** i for i in range(n - 1, -1, -1)], dtype=np.int32)

  # Precompute all priorities.
  priorities = np.array([priority(tuple(vector), n) for vector in all_vectors])

  # Build `capset` greedily, using priorities for prioritization.
  capset = np.empty(shape=(0, n), dtype=np.int32)
  while np.any(priorities != -np.inf):
    # Add a vector with maximum priority to `capset`, and set priorities of
    # invalidated vectors to `-inf`, so that they never get selected.
    max_index = np.argmax(priorities)
    vector = all_vectors[None, max_index]  # [1, n]
    blocking = np.einsum('cn,n->c', (- capset - vector) % 3, powers)  # [C]
    priorities[blocking] = -np.inf
    priorities[max_index] = -np.inf
    capset = np.concatenate([capset, vector], axis=0)

  return capset


@funsearch.evolve
def priority(el: tuple[int, ...], n: int) -> float:
  """Returns the priority with which we want to add `el` to the cap set."""
  return 0.0
```

**Figure C.6:** The full version of the program skeleton shown in Figure 2 (a), used to discover large cap sets.

## C.2 Admissible sets

In Figure 5 (b) we have seen a `priority` function discovered by *FunSearch* that not only yielded a full-size $\mathcal{I}(12, 7)$ admissible set, but also inspired us to conceive the notion of *symmetric* admissible sets (see Appendix D for more details). Here we show `priority` functions discovered by *FunSearch* that directly construct large *symmetric* admissible sets. See also Figure C.10 below for the program skeleton.

```python
def priority(el: tuple[int, ...], n: int, w: int) -> float:
  """Returns the priority with which we want to add `el` to the set."""
  score = 0.0
```

```python
  for i in range(n):
    if el[i] < el[i - 1]:
      score += 1
    elif el[i] < el[i - 2]:
      score += 0.05
    elif el[i] < el[i - 3]:
      score -= 0.05
    elif el[i] < el[i - 4]:
      score += 0.01
    elif el[i] < el[i - 5]:
      score -= 0.01
    elif el[i] < el[i - 6]:
      score += 0.001
    else:
      score += 0.005

  for i in range(n):
    if el[i] == el[i - 1]:
      score -= w
    elif el[i] == 0 and i != n - 1 and el[i + 1] != 0:
      score += w
    if el[i] != el[i - 1]:
      score += w

  for i in range(n):
    if el[i] < el[i - 1]:
      if el[i] == 0:
        score -= w
  return score
```

**Figure C.7:** A priority function that leads to a full-size constant-weight symmetric admissible set $\mathcal{I}(15, 10)$.

```python
def priority(el: tuple[int, ...], n: int, w: int) -> float:
  """Returns the priority with which we want to add `el` to the set."""
  score = 0
  coeff = 0
  for pos, x in zip(range(n), el):
    y = (el[(pos + 1) % n] - el[(pos)]) % n
    z = (el[(pos + 2) % n] - el[(pos)]) % n
    p = (el[(pos - 1) % n] + 1) % n

    u = (el[(pos - 2) % n] + 1) % n
    v = (el[(pos + 3) % n] + 1) % n

    score += 3 * p * (p + coeff) * (p + w) + (p + coeff)**2 * (w + 1)
    score += 2 * p * v * (p + w) + v * z * (-1 + w) - (p + coeff) * (-1 + w)
    score += v * (u + w) + u + 3 * u * y * (1 + w) + u * z * (w - 1) - (p + coeff) * (w - 1)
    score += (1 + w)**6 * 3 * coeff**2

  return score
```

**Figure C.8:** A priority function that leads to a constant-weight symmetric admissible set of size 43 596 in $\mathcal{A}(21, 15)$. The existence of such a set implies the cap set capacity lower bound of 2.2200.

```python
def priority(el: tuple[int, ...], n: int, w: int) -> float:
  """Returns the priority with which we want to add `el` to the set."""
  result = 0.0
  for i in range(n):
    n_violations = 0
    if el[i] < el[i - 1]:
      result += (el[i - 1] ** 0.5) * w ** 2 / (6 * 6)
      n_violations += 1
    if el[i] < el[i - 2]:
      result += el[i - 2] ** 0.5
```

```
        n_violations += 1
      if el[i - 1] != 0:
        result -= (el[i] - el[i - 1]) * w ** 2 / (6 * 3)
        n_violations += 2
      if el[i - 2] != 0:
        result -= (el[i] - el[i - 2]) * w ** 2 / (6 * 6) * (0.95 ** n_violations)
        n_violations += 1
      result -= (0.02 ** el[i]) * (el[i] - el[i - 8])
  return result
```

**Figure C.9:** A priority function that leads to a constant-weight symmetric admissible set of size 237 984 in $\mathcal{A}(24, 17)$. The existence of such a set implies the cap set capacity lower bound of 2.2202.

```
"""Finds large symmetric admissible sets."""
import itertools
import numpy as np

TRIPLES = [(0, 0, 0), (0, 0, 1), (0, 0, 2), (0, 1, 2), (0, 2, 1), (1, 1, 1), (2, 2, 2)]
INT_TO_WEIGHT = [0, 1, 1, 2, 2, 3, 3]


def expand_admissible_set(
    pre_admissible_set: list[tuple[int, ...]]) -> list[tuple[int, ...]]:
  """Expands a pre-admissible set into an admissible set."""
  num_groups = len(pre_admissible_set[0])
  admissible_set = []
  for row in pre_admissible_set:
    rotations = [[] for _ in range(num_groups)]
    for i in range(num_groups):
      x, y, z = TRIPLES[row[i]]
      rotations[i].append((x, y, z))
      if not x == y == z:
        rotations[i].append((z, x, y))
        rotations[i].append((y, z, x))
    product = list(itertools.product(*rotations))
    concatenated = [sum(xs, ()) for xs in product]
    admissible_set.extend(concatenated)
  return admissible_set


def get_surviving_children(extant_elements, new_element, valid_children):
  """Returns the indices of `valid_children` that remain valid after adding `new_element` to `extant_elements`."""
  bad_triples = set([
      (0, 0, 0), (0, 1, 1), (0, 2, 2), (0, 3, 3), (0, 4, 4), (0, 5, 5),
      (0, 6, 6), (1, 1, 1), (1, 1, 2), (1, 2, 2), (1, 2, 3), (1, 2, 4),
      (1, 3, 3), (1, 4, 4), (1, 5, 5), (1, 6, 6), (2, 2, 2), (2, 3, 3),
      (2, 4, 4), (2, 5, 5), (2, 6, 6), (3, 3, 3), (3, 3, 4), (3, 4, 4),
      (3, 4, 5), (3, 4, 6), (3, 5, 5), (3, 6, 6), (4, 4, 4), (4, 5, 5),
      (4, 6, 6), (5, 5, 5), (5, 5, 6), (5, 6, 6), (6, 6, 6)])

  # Compute.
  valid_indices = []
  for index, child in enumerate(valid_children):
    # Invalidate based on 2 elements from `new_element` and 1 element from a
    # potential child.
    if all(INT_TO_WEIGHT[x] <= INT_TO_WEIGHT[y]
           for x, y in zip(new_element, child)):
      continue
    # Invalidate based on 1 element from `new_element` and 2 elements from a
    # potential child.
    if all(INT_TO_WEIGHT[x] >= INT_TO_WEIGHT[y]
           for x, y in zip(new_element, child)):
      continue
    # Invalidate based on 1 element from `extant_elements`, 1 element from
    # `new_element`, and 1 element from a potential child.
    is_invalid = False
    for extant_element in extant_elements:
      if all(tuple(sorted((x, y, z))) in bad_triples
             for x, y, z in zip(extant_element, new_element, child)):
```

```
        is_invalid = True
        break
    if is_invalid:
      continue

    valid_indices.append(index)
  return valid_indices


def solve(n: int, w: int) -> list[tuple[int, ...]]:
  """Generates a symmetric constant-weight admissible set I(n, w)."""
  num_groups = n // 3
  assert 3 * num_groups == n

  # Compute the scores of all valid (weight w) children.
  valid_children = []
  for child in itertools.product(range(7), repeat=num_groups):
    weight = sum(INT_TO_WEIGHT[x] for x in child)
    if weight == w:
      valid_children.append(np.array(child, dtype=np.int32))
  valid_scores = np.array([
      priority(sum([TRIPLES[x] for x in xs], ()), n, w)
      for xs in valid_children])

  # Greedy search guided by the scores.
  pre_admissible_set = np.empty((0, num_groups), dtype=np.int32)
  while valid_children:
    max_index = np.argmax(valid_scores)
    max_child = valid_children[max_index]
    surviving_indices = get_surviving_children(pre_admissible_set, max_child,
                                               valid_children)
    valid_children = [valid_children[i] for i in surviving_indices]
    valid_scores = valid_scores[surviving_indices]

    pre_admissible_set = np.concatenate([pre_admissible_set, max_child[None]],
                                        axis=0)

  return expand_admissible_set(pre_admissible_set)


@funsearch.run
def evaluate(n: int, w: int) -> int:
  """Returns the size of the expanded admissible set."""
  return len(solve(n, w))


@funsearch.evolve
def priority(el: tuple[int, ...], n: int, w: int) -> float:
  """Returns the priority with which we want to add `el` to the set."""
  return 0.0
```

**Figure C.10:** The program skeleton used to search directly for symmetric admissible sets. See Appendix D for more details on this setting.

```
"""Finds large admissible sets."""
import itertools
import numpy as np


def block_children(scores: np.ndarray,
                   admissible_set: np.ndarray,
                   new_element: np.ndarray) -> None:
  """Modifies `scores` to -inf for elements blocked by `new_element`."""
  n = admissible_set.shape[-1]
  powers = np.array([3 ** i for i in range(n - 1, -1, -1)], dtype=np.int32)

  invalid_vals_raw = {
      (0, 0): (0,),
      (0, 1): (1,),
```

```
      (0, 2): (2,),
      (1, 0): (1,),
      (1, 1): (0, 1, 2),
      (1, 2): (1, 2),
      (2, 0): (2,),
      (2, 1): (1, 2),
      (2, 2): (0, 1, 2),
  }
  invalid_vals = [[np.array(invalid_vals_raw[(i, j)], dtype=np.int32)
                   for j in range(3)] for i in range(3)]

  # Block 2**w elements with the same support as `new_element`.
  w = np.count_nonzero(new_element)
  all_12s = np.array(list(itertools.product((1, 2), repeat=w)), dtype=np.int32)
  blocking = np.einsum('aw,w->a', all_12s, powers[new_element != 0])
  scores[blocking] = -np.inf

  # Block elements disallowed by a pair of an extant point and `new_element`.
  for extant_element in admissible_set:
    blocking = np.zeros(shape=(1,), dtype=np.int32)
    for e1, e2, power in zip(extant_element, new_element, powers):
      blocking = (blocking[:, None] + (invalid_vals[e1][e2] * power)[None, :]
                  ).ravel()
    scores[blocking] = -np.inf


def solve(n: int, w: int) -> list[tuple[int, ...]]:
  """Generates a constant-weight admissible set I(n, w)."""
  children = np.array(list(itertools.product((0, 1, 2), repeat=n)),
                      dtype=np.int32)

  scores = -np.inf * np.ones((3 ** n,), dtype=np.float32)
  for child_index, child in enumerate(children):
    if sum(child == 0) == n - w:
      scores[child_index] = priority(np.array(child), n, w)

  max_admissible_set = np.empty((0, n), dtype=np.int32)
  while np.any(scores != -np.inf):
    # Find element with largest score.
    max_index = np.argmax(scores)
    child = children[max_index]
    block_children(scores, max_admissible_set, child)
    max_admissible_set = np.concatenate([max_admissible_set, child[None]],
                                        axis=0)

  return [tuple(map(int, el)) for el in max_admissible_set]


@funsearch.run
def evaluate(n: int, w: int) -> int:
  """Returns the size of the constructed admissible set."""
  return len(solve(n, w))


@funsearch.evolve
def priority(el: tuple[int, ...], n: int, w: int) -> float:
  """Returns the priority with which we want to add `el` to the set."""
  return 0.0
```

**Figure C.11:** The program skeleton used to search for general (non-symmetric) admissible sets.

## C.3 Combinatorial optimization

```
def heuristic(item: float, bins: np.ndarray) -> np.ndarray:
  """Returns priority with which we want to add item to each bin.

  Args:
    item: Size of item to be added to the bin.
```

```
    bins: Array of capacities for each bin.

  Return:
    Array of same size as bins with priority score of each bin.
  """
  def s(bin, item):
    if bin - item <= 2:
      return 4
    elif (bin - item) <= 3:
      return 3
    elif (bin - item) <= 5:
      return 2
    elif (bin - item) <= 7:
      return 1
    elif (bin - item) <= 9:
      return 0.9
    elif (bin - item) <= 12:
      return 0.95
    elif (bin - item) <= 15:
      return 0.97
    elif (bin - item) <= 18:
      return 0.98
    elif (bin - item) <= 20:
      return 0.98
    elif (bin - item) <= 21:
      return 0.98
    else:
      return 0.99

  return np.array([s(bin, item) for bin in bins])
```

**Figure C.12:** Best performing heuristic for the OR datasets.

```
def heuristic(item: float, bins: np.ndarray) -> np.ndarray:
  """Returns priority with which we want to add item to each bin.

  Args:
    item: Size of item to be added to the bin.
    bins: Array of capacities for each bin.

  Return:
    Array of same size as bins with priority score of each bin.
  """
  score = 1.56 * bins - item - 4 * np.log(bins) + 0.16
  score[score > item] = item * 0.56
  return -score
```

**Figure C.13:** Simple heuristic for the OR datasets. While the performance of this heuristic is slightly worse than the best, it still significantly outperforms first fit and best fit across datasets.

```
def heuristic(item: float, bins: np.ndarray) -> np.ndarray:
  """Returns priority with which we want to add item to each bin.

  Args:
    item: Size of item to be added to the bin.
    bins: Array of capacities for each bin.

  Return:
    Array of same size as bins with priority score of each bin.
  """
  score = (bins - max(bins))**2 / item + bins**2 / item**2 + bins**2 / item**3
  score[bins > item] *= -1
  score[1:] -= score[:-1]
  return score
```

**Figure C.14:** Best performing discovered heuristic for the Weibull datasets.

## C.4 Shannon capacity of cycle graphs

See Appendix B.1 for a description of this problem, and the obtained results.

```
"""Obtains maximal independent sets."""
import funsearch
import itertools
import numpy as np


@funsearch.run
def evaluate(num_nodes: int, n: int) -> int:
  """Returns the size of an independent set."""
  independent_set = solve(num_nodes, n)
  return len(independent_set)


def solve(num_nodes: int, n: int) -> list[tuple[int, ...]]:
  """Gets independent set with maximal size.

  Args:
    num_nodes: The number of nodes of the base cyclic graph.
    n: The power we raise the graph to.

  Returns:
    A list of `n`-tuples in `{0, 1, 2, ..., num_nodes - 1}`.
  """
  to_block = np.array(list(itertools.product([-1, 0, 1], repeat=n)))

  # Powers in decreasing order for compatibility with `itertools.product`, so
  # that the relationship `i = children[i] @ powers` holds for all `i`.
  powers = np.array(
      [num_nodes ** i for i in range(n - 1, -1, -1)], dtype=np.int32)

  # Precompute the priority scores.
  children = np.array(
      list(itertools.product(range(num_nodes), repeat=n)), dtype=np.int32)
  scores = np.array([priority(tuple(child), num_nodes, n)
                     for child in children])

  # Build `max_set` greedily, using scores for prioritization.
  max_set = np.empty(shape=(0, n), dtype=np.int32)
  while np.any(scores != -np.inf):
    # Add a child with a maximum score to `max_set`, and set scores of
    # invalidated children to -inf, so that they never get selected.
    max_index = np.argmax(scores)
    child = children[None, max_index]  # [1, n]

    blocking = np.einsum(
        'cn,n->c', (to_block + child) % num_nodes, powers)  # [C]
    scores[blocking] = -np.inf
    max_set = np.concatenate([max_set, child], axis=0)

  return [tuple(map(int, el)) for el in max_set]


@funsearch.evolve
def priority(el: tuple[int, ...], num_nodes: int, n: int) -> float:
  """Returns the priority with which we want to add `el` to the set.

  Args:
    el: an n-tuple representing the element to consider whether to add.
    num_nodes: the number of nodes of the base graph.
    n: an integer, power of the graph.
```

```
    Returns:
      A number reflecting the priority with which we want to add `el` to the
      independent set.
    """
    return 0.
```

**Figure C.15:** User-provided problem specification for the Shannon capacity of cycle graphs problem (Appendix B.1). The initial `priority` function returns 0 regardless of the inputs.

```
def priority(el: tuple[int, ...], num_nodes: int, n: int) -> float:
    """Returns the priority with which we want to add `el` to the set.

    Args:
      el: an n-tuple representing the element to consider whether to add.
      num_nodes: the number of nodes of the base graph.
      n: an integer, power of the graph.

    Returns:
      A number reflecting the priority with which we want to add `el` to the
      independent set.
    """
    score = 0.
    for i in range(n):
      if el[i] == el[(i + 2) % n]:
        score += 1
      else:
        score -= 1
      x = ((n - 2) * el[i] - el[(i + 1) % n]
           - el[(i + 2) % n] - (n + 1) * el[(i + 3) % n]) % num_nodes
      score -= 0.5 * (x - el[(i + 1) % n]) ** 2
      score += 0.1 * (num_nodes - 1 - (x - 1) % num_nodes) ** 2
      score += 0.2 * (num_nodes - 1 - (x - 2) % num_nodes) ** 2
    return score
```

**Figure C.16:** A `priority` function that leads to an independent set of size 367 in $\mathcal{C}_7^{\boxtimes 5}$.

```
def priority(el: tuple[int, ...], num_nodes: int, n: int) -> float:
    """Returns the priority with which we want to add `el` to the set.

    Args:
      el: an n-tuple representing the element to consider whether to add.
      num_nodes: the number of nodes of the base graph.
      n: an integer, power of the graph.

    Returns:
      A number reflecting the priority with which we want to add `el` to the
      independent set.
    """
    s = 0.
    for i in range(n):
      s += el[i] << i
      s %= num_nodes
    return (2 * el[2] - 4 * el[0] + el[1]) % num_nodes + s
```

**Figure C.17:** A `priority` function that leads to independent sets of size 81, 324, 1458, 6561, and 26244 in $\mathcal{C}_9^{\boxtimes n}$, for $n = 3, 4, 5, 6, 7$, respectively.

```
def priority(el: tuple[int, ...], num_nodes: int, n: int) -> float:
    """Returns the priority with which we want to add `el` to the set.

    Args:
      el: an n-tuple representing the element to consider whether to add.
```

```
    num_nodes: the number of nodes of the base graph.
    n: an integer, power of the graph.

  Returns:
    A number reflecting the priority with which we want to add `el` to the
    independent set.
  """
  el_clipped = np.clip(el, a_min=None, a_max=num_nodes - 3)
  values = 2 * np.array(list(itertools.product(range(1, n), repeat=n)))
  multipliers = np.array(
      [num_nodes ** i for i in range(n - 1, -1, -1)], dtype=np.int32)
  x = np.sum((1 + values + el_clipped) * multipliers, axis=-1)
  return np.sum(x % (num_nodes - 2), dtype=float)
```

**Figure C.18:** A `priority` function that leads to an independent set of size 754 in $\mathcal{C}_{11}^{\boxtimes 4}$.

## C.5 Corners problem

For the corners problem (see Appendix B.2), we search for the largest set of indices in $\{0, 1, \ldots, 2^{2n}\}$ (for $\mathbb{Z}_2^n \times \mathbb{Z}_2^n$) or $\{0, 1, \ldots, 3^{2n}\}$ (for $\mathbb{Z}_3^n \times \mathbb{Z}_3^n$) satisfying the combinatorial degeneration constraints. These constraints are given in [27, Eq. 3]. For each candidate set of indices, we check if the combinatorial degeneration constraints are met, which can be done via a linear program that we implement in C++.

With *FunSearch*, we search for a `priority` function that we use to guide a greedy procedure, where we iteratively add the next valid node (index) with highest priority score, until it is not possible to add more valid nodes.

```
"""Corners problem via combinatorial degenerations."""
import corners
import funsearch
import itertools
import numpy as np


@funsearch.run
def evaluate(n: int) -> int:
  """Returns the size of the maximum set of indices found by DFS."""
  return len(solve(n))


def solve(n: int) -> list[tuple[int, ...]]:
  """Runs DFS to find a large set of indices."""
  # Obtain the priority scores.
  scores = np.array(
      [priority(el, n) for el in itertools.product(range(2), repeat=2 * n)]
  )
  all_indices = np.arange(len(scores), dtype=np.int32)
  # Run a greedy a approach that iteratively adds the next highest-priority
  # index that guarantees the combinatorial degeneration property.
  return corners.greedy(all_indices, scores)


@funsearch.evolve
def priority(el: tuple[int, ...], n: int) -> float:
  """Returns the priority with which we want to add `el`.

  Args:
    el: A candidate element to be considered, as a tuple of length `2 * n` with
      elements in {0, 1}.
    n: Power of the graph.

  Returns:
    A number reflecting the priority with which we want to add `el` to the set.
```

```
    """
    return 0.
```

**Figure C.19:** User-provided problem specification for the corners problem (Appendix B.2) in $\mathbb{Z}_2^n \times \mathbb{Z}_2^n$ (it is analogous for the case $\mathbb{Z}_3^n \times \mathbb{Z}_3^n$). The initial `priority` function returns 0 regardless of the inputs.

```
def priority(el: tuple[int, ...], n: int) -> float:
  """Returns the priority with which we want to add `el`.

  Args:
    el: A candidate element to be considered, as a tuple of length `2 * n` with
      elements in {0, 1}.
    n: Power of the graph.

  Returns:
    A number reflecting the priority with which we want to add `el` to the set.
  """
  s0 = 0
  s1 = 0
  for i in range(1, 2 * n - 1):
    cur = el[i] ^ el[i - 1] ^ el[i + 1]
    if i < n - 1:
      if cur != 1:
        s0 += 1
    if i > n:
      if cur != 1:
        s1 += 1
  t1 = - ((s1 - s0) ** 6 - 3 * (s1 - s0) ** 3 + (s1 - s0))
  t2 = (el[0] + el[2 * n - 1]) ** 6
  t3 = - 3 * (el[0] + el[2 * n - 1]) ** 3
  t4 = el[0] + el[2 * n - 1]
  t5 = 0.01 * (
      el[0] ^ el[n - 1] + el[0] ^ el[2 * n - 1] - el[n - 1] ^ el[2 * n - 1])
  t6 = 0.04 * min(el[n - 1], el[n]) ** 6
  return t1 + t2 + t3 + t4 + t5 + t6
```

**Figure C.20:** A `priority` function that leads to a set of indices of size 137 satisfying the combinatorial degeneration constraints for $\mathbb{Z}_2^n \times \mathbb{Z}_2^n$ when $n = 4$.

```
def priority(el: tuple[int, ...], n: int) -> float:
  """Returns the priority with which we want to add `el`.

  Args:
    el: A candidate element to be considered, as a tuple of length `2 * n` with
      elements in {0, 1, 2}.
    n: Power of the graph.

  Returns:
    A number reflecting the priority with which we want to add `el` to the set.
  """
  def aux_fn(a: float) -> float:
    return (int(np.absolute(a) // 3) + 1) * 3 - np.absolute(a)

  num = 0
  m = 0
  c = 0
  s = 0
  for i, (a, b) in enumerate(itertools.zip_longest(el, el[1:], fillvalue=3)):
    if i % 2:
      continue
    s += max(0, np.absolute(a - b) - 1)
    num += aux_fn(a + b)
    m += aux_fn((a + b) / 3)
    c += aux_fn(a - b)
```

```
    s += (num // 3 + m) ** 2 / 3 + (num + m) ** 2 - (num + m + c) ** 2
  return -s / (1 + max(0.0, (num - 1) // 3))
```

**Figure C.21:** A `priority` function that leads to a set of indices of size 53 satisfying the combinatorial degeneration constraints for $\mathbb{Z}_3^n \times \mathbb{Z}_3^n$ when $n = 2$.

```
def priority(el: tuple[int, ...], n: int) -> float:
  """Returns the priority with which we want to add `el`.

  Args:
    el: A candidate element to be considered, as a tuple of length `2 * n` with
      elements in {0, 1, 2}.
    n: Power of the graph.

  Returns:
    A number reflecting the priority with which we want to add `el` to the set.
  """
  t = 0
  t2 = 0
  x = 0
  x2 = 0
  for i in range(n):
    t += el[2 * i] + el[2 * i + 1]
    if el[2 * i] > el[2 * i + 1]:
      t += 1
    if el[2 * i + 1] == el[2 * i]:
      t2 += 2
    else:
      t2 -= 1
    if i > 0 and (el[2 * i - 1] == el[2 * i] == 2):
      t2 += 1
    if i < n - 1 and (el[2 * (i + 1)] == el[2 * i] == 2):
      t2 += 1
    x += el[2 * i]
    x2 += el[2 * i] ** 2
  return -t + 2 * t2 + 0.1 * el[-1] - 2 * (n - x) + n - 2. + 2 * t / n - x2 / n
```

**Figure C.22:** A `priority` function that leads to a set of indices of size 370 satisfying the combinatorial degeneration constraints for $\mathbb{Z}_3^n \times \mathbb{Z}_3^n$ when $n = 3$.

# D Symmetric admissible sets and pre-admissible sets

This section formally describes the notion of *symmetric* admissible sets, a notion which was inspired by inspecting the source code discovered by *FunSearch* for constructing an $\mathcal{I}(12, 7)$ admissible set (see Appendix E.3 for more details). Afterwards this section describes how we can directly search for symmetric admissible sets, by searching for "generators" of symmetric admissible sets – we call these generators *pre-admissible sets*. This is the method we used to obtain our best lower bounds on the cap set capacity.

First we recall the definition of an *admissible set*, as it appears in [6]. Note that this definition can be traced back to [29], which however used a different nomenclature.

**Definition 1.** A set $A \subseteq \{0, 1, 2\}^n$ is called *admissible* if

1. for all *ordered* pairs of distinct vectors $(\mathbf{x}, \mathbf{y}) \in A^2$ there is a coordinate $1 \leq i \leq n$ such that $x_i = 0 \neq y_i$,
2. for all triples of distinct vectors $\mathbf{x}, \mathbf{y}, \mathbf{z} \in A$ there is a coordinate $1 \leq i \leq n$ such that

$$\{x_i, y_i, z_i\} \in \{\{0, 1, 2\}, \{0, 0, 1\}, \{0, 0, 2\}\}, \tag{1}$$

where $\{\cdot,\cdot,\cdot\}$ is used to denote a multiset of three elements here.

The following definition is constructed by us, after being inspired by one of the functions discovered by *FunSearch*.

**Definition 2.** We call an admissible set $A \subseteq \{0,1,2\}^n$ in $n = 3k$ dimensions *symmetric* if it is preserved (as a set, i.e. up to reordering) under all $3^k$ cyclic permutations of coordinates within the $k$ disjoint consecutive triples of coordinates $[0,1,2], \ldots, [3(k-1), 3(k-1)+1, 3(k-1)+2]$. In other words, it is preserved as a set under the group action $\mathbb{Z}_3^k$, with the action $f_c$ of a group element $g = (g_1, \ldots, g_k) \in \mathbb{Z}_3^k$ on a vector $\mathbf{x} \in A$ given by

$$\begin{aligned} f_c(g, \mathbf{x}) &= f_c((g_1, \ldots, g_k), (x_0, x_1, x_2, \ldots, x_{3(k-1)+0, 3(k-1)+1, 3(k-1)+2})) \\ &= (x_{g_1(0)}, x_{g_1(1)}, x_{g_1(2)}, \ldots, x_{3(k-1)+g_{k-1}(0)}, x_{3(k-1)+g_{k-1}(1)}, x_{3(k-1)+g_{k-1}(2)}), \end{aligned} \tag{2}$$

where $g_i(j) = (i+j) \bmod 3$.

*Remark* 1. Inspecting the definition of admissible sets, it is easy to see that if we arbitrarily permute (relabel) the $n$ coordinates of a valid admissible set $A \subseteq \{0,1,2\}^n$, the resulting set is still admissible. However, Definition 2 of *symmetric* admissible set states a stronger requirement – under specific permutations of coordinates, the resulting admissible set must remain the *same* (as a set, i.e. up to reordering of its $A$ elements).

We'll be searching directly for *symmetric* admissible sets. To set up this restricted search space, we'll make use of the following definitions.

**Definition 3.** Let $\mathcal{S} := \{0,1,\ldots,6\}$ and define a *decoding function* $\phi : \mathcal{S} \to \{0,1,2\}^3$ as

$$\phi(a) = \begin{cases} (0,0,0) & \text{if } a = 0 \\ (0,0,1) & \text{if } a = 1 \\ (0,0,2) & \text{if } a = 2 \\ (0,1,2) & \text{if } a = 3 \\ (0,2,1) & \text{if } a = 4 \\ (1,1,1) & \text{if } a = 5 \\ (2,2,2) & \text{if } a = 6 \end{cases} \tag{3}$$

**Definition 4.** The *orbit* of $(x,y,z) \in \mathrm{Im}(\phi)$ is the (deduplicated) set

$$O((x,y,z)) := \{(x,y,z), (y,z,x), (z,x,y)\}, \tag{4}$$

i.e. the orbit of $(x,y,z)$ under the action of cyclically permuting its elements. The *orbit length* $l : \mathrm{Im}(\phi) \to \{1,3\}$ is defined to be the size of this set:

$$l(\phi(a)) := |O(\phi(a))| = \begin{cases} 1 & \text{if } a \in \{0,5,6\} \\ 3 & \text{if } a \in \{1,2,3,4\} \end{cases} \tag{5}$$

*Remark* 2. The decoded elements $\mathrm{Im}(\phi)$ are precisely the elements of $\{0,1,2\}^3$ (up to cyclic permutations) whose orbits form a valid admissible set. For example, $(0,0,2)$ generates the admissible set $\{(0,0,2),(0,2,0),(2,0,0)\}$, whereas $\{1,1,2\} \notin \mathrm{Im}(\phi)$ would generate the set $\{(1,1,2),(1,2,1),(2,1,1)\}$, which is not admissible.

**Definition 5.** The *weight* of an element in $\mathcal{S} = \{0, 1, \dots, 6\}$ is given by $w : \mathcal{S} \to \{0, 1, 2, 3\}$ defined as

$$w(a) = \#\{\text{nonzeros in } \phi(a)\} = \begin{cases} 0 & \text{if } a = 0 \\ 1 & \text{if } a \in \{1, 2\} \\ 2 & \text{if } a \in \{3, 4\} \\ 3 & \text{if } a \in \{5, 6\} \end{cases} \tag{6}$$

**Definition 6.** For $k \in \mathbb{N}$, a $k$-dimensional *pre-admissible set* is a set $P \subseteq \{0, 1, \dots, 6\}^k$ such that

1. for any *ordered* pair of distinct vectors $(\mathbf{x}, \mathbf{y}) \in P^2$ there is a column $1 \le i \le k$ such that $w(x_i) < w(y_i)$,
2. for any triple of distinct vectors $\mathbf{x}, \mathbf{y}, \mathbf{z} \in P$ there is a column $1 \le i \le k$ such that

$$\begin{aligned} \{x_k, y_k, z_k\} \in \{&\{0,0,1\}, \{0,0,2\}, \{0,0,3\}, \{0,0,4\}, \{0,0,5\}, \{0,0,6\}, \{0,1,2\}, \{0,1,3\}, \{0,1,4\}, \\ &\{0,1,5\}, \{0,1,6\}, \{0,2,3\}, \{0,2,4\}, \{0,2,5\}, \{0,2,6\}, \{0,3,4\}, \{0,3,5\}, \{0,3,6\}, \\ &\{0,4,5\}, \{0,4,6\}, \{0,5,6\}, \{1,1,3\}, \{1,1,4\}, \{1,1,5\}, \{1,1,6\}, \{1,2,5\}, \{1,2,6\}, \\ &\{1,3,4\}, \{1,3,5\}, \{1,3,6\}, \{1,4,5\}, \{1,4,6\}, \{1,5,6\}, \{2,2,3\}, \{2,2,4\}, \{2,2,5\}, \\ &\{2,2,6\}, \{2,3,4\}, \{2,3,5\}, \{2,3,6\}, \{2,4,5\}, \{2,4,6\}, \{2,5,6\}, \{3,3,5\}, \{3,3,6\}, \\ &\{3,5,6\}, \{4,4,5\}, \{4,4,6\}, \{4,5,6\}\} \end{aligned} \tag{7}$$

where $\{\cdot, \cdot, \cdot\}$ is used to denote a multi-set of 3 elements here.

As the naming suggests, the point of a pre-admissible set is that the orbits of its elements automatically yield an admissible set. This is captured by the following theorem.

**Theorem D.1.** *Given a $k$-dimensional pre-admissible set $P$, the set*

$$A := \bigcup_{\mathbf{x} \in P} \bigotimes_{i=1}^{k} O(\phi(x_k)) \tag{8}$$

*is a* symmetric *admissible set in $n = 3k$ dimensions, of size $\sum_{\mathbf{x} \in P} \prod_{i=1}^{k} l(x_i)$.*

*Remark* 3. In the statement of Theorem D.1 we slightly abuse notation by identifying elements of $\bigotimes_{i=1}^{k} O(\phi(x_k)) \subseteq (\{0, 1, 2\}^3)^k$ with the corresponding flattened elements of $\{0, 1, 2\}^{3k}$.

*Remark* 4. Theorem D.1 works for general admissible sets, not just constant-weight.

*Proof of Theorem D.1.* We need to verify the two defining conditions of an admissible set. Before that, we start with a simple observation: since $P$ is a set, it doesn't contain duplicates, and therefore it is easy to verify that the union in the definition of $A$ is a disjoint union. In particular, each vector $\mathbf{x} \in A$ stems from a single (well-defined) element $\hat{\mathbf{x}} \in P$, which we call its *generator.*

**Condition 1** Suppose $\mathbf{x}, \mathbf{y}$ is an ordered pair of vectors from $A$. There are two cases we need to consider:

1. $\mathbf{x}, \mathbf{y}$ come from the same generator $\hat{\mathbf{x}} \in P$

   Since $\mathbf{x}, \mathbf{y}$ are distinct, there must be a coordinate $1 \le i \le k$ in $\hat{\mathbf{x}}$ such that $\mathbf{x}_{3i:3(i+1)} \ne \mathbf{y}_{3i:3(i+1)}$. In this case $l(\hat{x}_i) \ne 1$, so $\hat{x}_i \in \{1, 2, 3, 4\}$, and it is easy to check that one of $j \in \{3i, 3i+1, 3i+2\}$ must be such that $x_j = 0 \ne y_j$.

2. $\mathbf{x}, \mathbf{y}$ come from different generators $\hat{\mathbf{x}}, \hat{\mathbf{y}} \in P$

   By the first defining condition of pre-admissible set, there is a coordinate $1 \le i \le k$ such that $w(\hat{x}_i) < w(\hat{y}_i)$. This means there are strictly more zero coordinates in $\phi(\hat{x}_i)$ than in $\phi(\hat{y}_i)$, which implies that at least one of $j \in \{3i, 3i+1, 3i+2\}$ is such that $x_j = 0 \neq y_j$.

**Condition 2** Suppose $\mathbf{x}, \mathbf{y}, \mathbf{z}$ is a triple of distinct vectors from $A$. Without loss of generality, there are just three cases we need to consider:

1. $\mathbf{x}, \mathbf{y}, \mathbf{z}$ come from the same generator $\hat{\mathbf{x}} \in P$

   Since $\mathbf{x}, \mathbf{y}, \mathbf{z}$ are distinct, there must be a coordinate $1 \le i \le k$ in $\hat{\mathbf{x}}$ such that $\mathbf{x}_{3i:3(i+1)}$, $\mathbf{y}_{3i:3(i+1)}$, and $\mathbf{z}_{3i:3(i+1)}$ are not all the same. In this case $l(\hat{x}_i) \neq 1$, so $\hat{x}_i \in \{1, 2, 3, 4\}$, and it is easy to check that one of $j \in \{3i, 3i+1, 3i+2\}$ must be such that $\{x_j, y_j, z_j\}$ equals one of $\{\{0, 0, 1\}, \{0, 0, 2\}, \{0, 1, 2\}\}$ as a multi-set.

2. $\mathbf{x}, \mathbf{y}$ come from the same generator $\hat{\mathbf{x}} \in P$, and $\mathbf{z}$ comes from a different generator $\hat{\mathbf{z}} \in P$

   By the first defining condition of pre-admissible set, there is a coordinate $1 \le i \le k$ such that $w(\hat{x}_i) < w(\hat{z}_i)$. This means there are strictly more zero coordinates in $\phi(\hat{x}_i)$ than in $\phi(\hat{z}_i)$. A simple case analysis then shows that one of $j \in \{3i, 3i+1, 3i+2\}$ must be such that $\{x_j, y_j, z_j\}$ equals one of $\{\{0, 0, 1\}, \{0, 0, 2\}, \{0, 1, 2\}\}$ as a multi-set. Specifically:

   - $w(\hat{z}_i) = 0$ is impossible as $0 \le w(\hat{x}_i) < w(\hat{z}_i)$;
   - $w(\hat{z}_i) = 1$ implies that $\hat{x}_i = 0$ and $\hat{z}_i \in \{1, 2\}$, so there will be a coordinate with values $\{0, 0, 1\}$ or $\{0, 0, 2\}$;
   - $w(\hat{z}_i) = 2$ implies that $\hat{x}_i \in \{0, 1, 2\}$ and $\hat{z}_i \in \{3, 4\}$, and it is easy to check that there will be a coordinate with values $\{0, 0, 1\}$, $\{0, 0, 2\}$, or $\{0, 1, 2\}$;
   - $w(\hat{z}_i) = 3$ implies that $\hat{x}_i \in \{0, 1, 2, 3, 4\}$ and $\hat{z}_i \in \{5, 6\}$. If $\mathbf{x}_{3i:3(i+1)}$ and $\mathbf{y}_{3i:3(i+1)}$ both equal 0 in some coordinate $j$, in that coordinate we have $\{x_j, y_j, z_j\} \in \{\{0, 0, 1\}, \{0, 0, 2\}\}$. Otherwise we will always have a coordinate in which $\{x_j, y_j, z_j\} = \{0, 1, 2\}$.

3. $\mathbf{x}, \mathbf{y}, \mathbf{z}$ come from different generators $\hat{\mathbf{x}}, \hat{\mathbf{y}}, \hat{\mathbf{z}} \in P$

   By the second defining condition of pre-admissible set, there is a coordinate $1 \le i \le k$ such that $\{\hat{x}_k, \hat{y}_k, \hat{z}_k\}$ equals (as a multi-set) one of the 49 possibilities in Equation 7. It is a routine (but tedious unless automated) check to verify that in each of the 49 possibilities, taking any three elements from the three respective orbits guarantees a coordinate whose elements equal one of $\{\{0, 0, 1\}, \{0, 0, 2\}, \{0, 1, 2\}\}$ as a multi-set.

□

# E More details

## E.1 Hyperparameters

Table E.2 provides the values of all the hyperparameters in *FunSearch*. Only the evaluator parameters are problem-dependent, as evaluating a candidate solution requires a different amount of resources in different problems. The values of evaluator hyperparameters provided in Table E.2 are the default values used for the cap set problem; we set some of them differently for some of the other problems:

- For admissible sets, we set the timeout to 120 seconds and the memory limit to 5 GB.
- For bin packing, the timeout is 300 seconds and the memory limit is 5 GB.
- For the problem of Shannon capacity of cycle graphs (Appendix B.1), the timeout is 90 seconds. For the experiments on $\mathcal{C}_9$, the timeout is 900 seconds, the memory limit is 64 GB, and the number of evaluators is 280.
- For the corners problem (Appendix B.2), the timeout is 200 seconds and the number of evaluators is 420.

| | Hyperparameter | Value |
|---|---|---|
| **Samplers** | Number of samplers | 15 |
| | Number of generated samples per prompt | 4 |
| | LLM sampling temperature | 1.0 |
| | LLM nucleus sampling probability | 0.95 |
| **Evaluators** | Number of evaluators | 140 |
| | Timeout (in seconds) | 30 |
| | Memory limit (in GB) | 2 |
| **Evolutionary algorithm** | Number of previous programs per prompt ($k$ in main paper) | 2 |
| | Number of islands ($m$ in Appendix A.1) | 10 |
| | Reset time for islands (in hours) | 4 |
| | Initial temperature for cluster probabilities ($T_0$ in Eq. 1) | 0.1 |
| | Temperature period for cluster probabilities ($N$ in Eq. 1) | 30 000 |
| | Temperature for program probabilities ($T_{\text{program}}$ in Appendix A.1) | 1.0 |

**Table E.2:** *FunSearch* hyperparameters.

## E.2 Explicit construction of a size-512 cap set in $\mathbb{Z}_3^8$

Once *FunSearch* discovered a priority function $\mathbb{Z}_3^8 \to \mathbb{R}$ that leads to a size-512 cap set in $\mathbb{Z}_3^8$ (see Figure 4 b) we also looked at the cap set itself, as a sequence of 512 length-8 vectors over $\mathbb{Z}_3$. To our surprise this set of vectors exhibited some obvious regularities: most apparently, the first 128 vectors contained no 0 entries. Note that a cap set is in principle an unordered set, and it is only thanks to searching in function space that in the particular ordering found by *FunSearch* these full-weight vectors appeared as the first 128 entries, rather than being scattered throughout the set. This initial observation prompted us to analyse the set further. It was straightforward to determine that the aforementioned 128 full-weight vectors were followed by 256 vectors of weight 4, and the set was completed by 128 vectors of weight 5 (the weight of a vector is its number of nonzero entries).

In all three cases $w \in \{8, 4, 5\}$ of weight-$w$ vectors, the cap set does not contain all possible vectors of that weight; for example it contains precisely half of all possible full weight ($w = 8$) vectors. It was a natural question to understand what half this is. If we only had the set of 128 vectors to look at, this might be a more difficult question to answer (likely requiring open-ended exploratory analysis on these vectors to understand which are included and which are not). However, we also had the option to refer back to the `priority` function discovered by *FunSearch* in order to get a hint of what rules are being used in determining which vector to include and which to exclude. (This is only complicated by the fact that we also exclude high-priority vectors that would invalidate the cap set). The source code of Figure 4 (b) reveals that the priority of a vector `el` heavily depends on which of the “reflection” equalities `el[1] == el[-1]`, `el[2] == el[-2]`, `el[3] == el[-3]` hold. This prompted us to introduce the notion of *reflections*, which counts how many of these equalities

hold, and we found that the cap set contains precisely those full-weight vectors that have at least two reflections.

We then noticed that the notion of *reflections* is also useful to describe which vectors are included in the weight-4 and weight-5 portions of the cap set, although in those cases additional conditions needed to be added (we obtained these by further analysing the included vectors). Figure 4 (c) shows the resulting stand-alone function that explicitly describes the 512 vectors that form a cap set in $\mathbb{Z}_3^8$.

The construction reveals that the cap set can be constructed as the union of four 128-element sets, each with a different defining property. For example, the first set contains a specific half of all vectors without a 0 entry, whereas the second set contains all vectors whose nonzero entries appear in 4 specific coordinates. These ideas are strikingly similar to the construction of the Hill cap [30, 31], which results in the optimal 112-cap in $\mathbb{Z}_3^6$. See Figure E.23 for a comparison of these constructions in source code space. This observation offers the prospect of studying whether constructions like these generalize to higher dimensions $n$.

```python
def get_capset(n: int) -> list[tuple[int, ...]]:
  """Build a size-112 cap set in n=6 dimensions."""
  V = np.array(list(itertools.product(range(3),
  ↪   repeat=n)), dtype=np.int32)
  reflections = lambda v: sum(1 for i in range(n //
  ↪   2) if v[i] == v[n - 1 - i])

  # First we list 2^(n-1) full-weight vectors with 1
  ↪   or 3 reflections.
  full_support_vectors = [
      v for v in V
      if np.count_nonzero(v) == n  # Full weight.
      and reflections(v) in [1, 3]]

  # Then we list half-support vectors with allowed
  ↪   support.
  allowed_supports = [
      (0, 1, 2), (0, 1, 3), (0, 2, 4), (0, 3, 5),
      (0, 4, 5), (1, 2, 5), (1, 3, 4), (1, 4, 5),
      (2, 3, 4), (2, 3, 5)]
  half_support_vectors = [
      v for v in V
      if tuple(i for i in range(n) if v[i] != 0) in
      ↪   allowed_supports]

  return full_support_vectors + half_support_vectors
```

```python
def get_capset(n: int) -> list[tuple[int, ...]]:
  """Build a size-512 cap set in n=8 dimensions."""
  V = np.array(list(itertools.product(range(3),
  ↪   repeat=n)), dtype=np.int32)
  reflections = lambda v: sum(1 for i in range(1, n
  ↪   // 2) if v[i] == v[-i])

  # First we list 2^(n-1) full-weight vectors with 2
  ↪   or 3 reflections.
  full_support_vectors = [
      v for v in V
      if np.count_nonzero(v) == n  # Full weight.
      and reflections(v) in [2, 3]]

  # Then we list half-support vectors with allowed
  ↪   support and <= 1 reflections.
  allowed_supports = [
      (0, 1, 2, 3), (0, 1, 2, 5), (0, 1, 2, 7),
      (0, 1, 2, 6), (0, 1, 3, 7), (0, 1, 6, 7),
      (0, 3, 6, 7), (0, 5, 6, 7), (0, 1, 5, 7),
      (1, 3, 4, 6), (1, 4, 5, 6), (0, 2, 3, 6),
      (2, 3, 4, 7), (2, 4, 5, 7), (0, 2, 6, 7),
      (0, 2, 5, 6), (1, 2, 4, 7), (1, 2, 4, 6),
      (1, 3, 4, 7), (1, 4, 6, 7), (1, 4, 5, 7),
      (2, 3, 4, 6), (2, 4, 6, 7), (2, 4, 5, 6)]
  half_support_vectors = [
      v for v in V
      if tuple(i for i in range(n) if v[i] != 0) in
      ↪   allowed_supports
      and reflections(v) <= 1]

  # Finally we add 128 weight-5 vectors with <= 1
  ↪   reflections.
  allowed_zeros = [
      (0, 4, 7), (0, 2, 4), (0, 1, 4), (0, 4, 6),
      (1, 2, 6), (2, 6, 7), (1, 2, 7), (1, 6, 7)]
  weight5_vectors = [
      v for v in V
      if np.count_nonzero(v) == 5  # Weight is 5.
      and tuple(i for i in range(n) if v[i] == 0) in
      ↪   allowed_zeros
      and reflections(v) <= 1
      and (v[1]*v[7]) % 3 != 1 and (v[2]*v[6]) % 3
      ↪   != 1]

  return full_support_vectors + half_support_vectors
  ↪   + weight5_vectors
```

**Figure E.23:** Juxtaposition of explicit constructions of largest known cap sets in $n = 6$ dimensions (left) and $n = 8$ dimensions (right). The construction on the left was hand-constructed based on descriptions of the Hill cap in the literature [31]. The cap set of size 512 in $n = 8$ dimensions was discovered by *FunSearch*, and the cleaned up explicit construction showed on the right was obtained by us through the process described in Appendix E.2.

## E.3 Conception of symmetric admissible sets

```python
def priority(el: tuple[int, ...], n: int, w: int) -> float:
  score = 0.0
  for i in range(n):
    if el[i] == 1:
      score -= 0.9 ** (i % 4)
    if el[i] == 2:
      score -= 0.98 ** (30 - (i % 4))
    if el[i] == 1 and el[i - 4] == 1:
      score -= 0.98 ** (30 - (i % 4))
    if el[i] == 2 and el[i - 4] != 0:
      score -= 0.98 ** (30 - (i % 4))
  return score
```

**(a)** First discovered function.

```python
def priority(el: tuple[int, ...], n: int, w: int) -> float:
  score = 0.0
  for i in range(n):
    if el[i] == 1:
      score -= 0.9 ** (i % 4)
    if el[i] == 2:
      score -= 0.98 ** (30 - (i % 4))
    if el[i] == 1 and el[i - 4] == 1:
      score -= 0.98 ** (30 - (i % 4))
    if el[i] == 2 and el[i - 4] != 0:
      score -= 0.98 ** (30 - (i % 4))
    if el[i] == 2 and el[i - 4] == 1 and el[i - 8] == 2:
      score -= 0.98 ** (30 - (i % 4))
      score -= 6.3
    if el[i] == 2 and el[i - 4] == 2 and el[i - 8] == 1:
      score -= 0.98 ** (30 - (i % 4))
    if el[i] == 2 and el[i - 4] == 1 and el[i - 8] == 1:
      score -= 6.3
    if el[i] == 2 and el[i - 4] == 0 and el[i - 8] == 2:
      score -= 6.3
    if el[i] == 1 and el[i - 4] == 1 and el[i - 8] == 0:
      score -= 2.2
  return score
```

**(b)** Further evolved to increase interpretabilty.

**Figure E.24:** Two `priority` functions discovered by *FunSearch*, both of which yield a full-size $\mathcal{I}(12, 7)$ admissible set. The function on the left was discovered first. Afterwards we used *FunSearch* to evolve this function further, using a modified scoring function that we hypothesised would lead to better interpretability. This resulted in the function shown on the right. This longer function reveals more clearly the regularities of how the function uses the loop index `i` and which entries of `el` are accessed jointly.

Once *FunSearch* discovered a `priority` function $\{0, 1, 2\}^{12} \to \mathbb{R}$ that leads to a full-size $\mathcal{I}(12, 7)$ admissible set $A$ (see Figure E.24a), we were curious to understand what this function is doing. We proceeded in two interconnected ways. First, we visually inspected the source code, and started noticing a few interesting features (more on these below). Second, we leveraged the flexibility of *FunSearch* to optimise an arbitrary numerical property of the program, and used *FunSearch* to further evolve the discovered program in such a way that it becomes more *interpretable*. Specifically, we quantified interpretability as the negative of the number of high-scoring elements `el` that do *not* get included in the constructed admissible set $A$ (because they would violate a defining condition of admissible set). More formally, the interpretability score $s_I(A)$ and total score $s(A)$ of a program producing an admissible set $A$ in this modified setting was

$$s_I(A) := \left|\left\{\texttt{el} \in \{0,1,2\}^n \mid \texttt{el} \notin A \land \exists x \in A \text{ s.t. } \texttt{priority}(x, n, w) < \texttt{priority}(\texttt{el}, n, w)\right\}\right| \quad (9)$$

$$s(A) := |A| - 10^{-6} s_I(A) \quad (10)$$

The intuition was that a priority function that describes the admissible set $A$ more tightly, relying less on filtering away high-scoring but invalid elements, would be more interpretable. The resulting function is shown in Figure E.24b, and visually inspecting it has helped us notice more easily the following features of this program:

1. As the function loops through the $n$ coordinates `i` $\in \{0, 1, \ldots, 11\}$, apart from indexing into the vector `el` the value of `i` is only accessed through its modulus `i % 4`, which is constant within each of the four coordinate groups $\{0, 4, 8\}$, $\{1, 5, 9\}$, $\{2, 6, 10\}$, $\{3, 7, 11\}$.
2. Each `if` condition in the function accesses between one and three entries of `el`, and thanks to how negative indices wrap around in Python, the set of accessed elements is always contained in one of the four coordinate groups $\{0, 4, 8\}$, $\{1, 5, 9\}$, $\{2, 6, 10\}$, $\{3, 7, 11\}$.
3. Since the priority is computed by `i` looping through all $n$ coordinates, the resulting priority is actually invariant under cyclically permuting entries of `el` within any of the aforementioned four coordinate groups (cf. Appendix D).

The final observation may not automatically guarantee that our discovered $\mathcal{I}(12, 7)$ admissible set $A$ is invariant under cyclically permuting coordinates within each four coordinate groups, as some high-priority elements could still potentially get skipped due to violating the admissible set condition, and thus breaking the invariance. However, this turned out not to be the case here — we verified that $A$ is in fact invariant under this symmetry, which we formalized as Definition 2, calling admissible sets possessing this invariance *symmetric*.

We then hypothesised that large *symmetric* admissible sets may exist for other values of $n = 3k$ and $1 \leq w < n$. We modified the program skeleton in the program specification that we pass to *FunSearch* such that it directly searches for symmetric admissible sets only (see Figure C.10). This is a more restricted but also much smaller search space, and we quickly discovered that symmetric admissible sets do in fact exist for all choices of $n \in \{3, 6, 9, 12\}$ and each $1 \leq w < n$. This encouraged us to try searching for even larger symmetric admissible sets, resulting in a full-size $\mathcal{I}(15, 10)$ and a non-full-size admissible set in $\mathcal{A}(21, 15)$. These imply further improvements to the lower bound on the cap set capacity, as described in the main paper.

## E.4 Bin packing datasets

**OR-Library datasets.** We evaluate *FunSearch* on the well-known OR-Library bin packing benchmarks [32], using the binpack1, binpack2, binpack3 and binpack4 datasets, each containing 20 bin packing instances, with 120, 250, 500, and 1 000 items, respectively. These instances were generated by sampling item sizes uniformly from the interval $[20, 100]$. For all datasets, the capacity of the bins is set to 150. To evolve a heuristic with *FunSearch*, we generated a training dataset of 20 instances each with 120 items sampled from $[20, 100]$ (similarly to the binpack1 instances). To evaluate our heuristic during training, we also generated a validation dataset of 20 instances of 250 items sampled from $[20, 100]$ (similarly to the binpack2 instances). We then select the best heuristics with respect to the validation dataset and test them on the binpack1 to binpack4 instances.

**Weibull datasets.** The Weibull datasets were generated by sampling from a Weibull$(45, 3)$ distribution. The parameters of this distribution were chosen based on standard values in the literature [33]. We further clipped the samples at 100 and rounded all items sizes to the nearest integer in $\{1, 2, \ldots, 100\}$. We generated a training dataset containing 5 instances each with 5 000 items and a validation dataset with the same number of instances and items. To test our learned heuristics, we generated test sets of 5 instances with 5 000 items, 5 instances of 10 000 items and 1 instance of 100 000 items (referred to as Weibull 5k, Weibull 10k and Weibull 100k respectively).

### E.5 Bin packing visualizations

As noted in the main paper, instead of packing items into bins with the least capacity (like best fit), the *FunSearch* heuristics typically assign items to least capacity bins only if the fit is very tight after placing the item. Otherwise, the item is placed in another bin which would leave more space after the item is placed. This strategy avoids leaving small gaps in bins that are unlikely to ever be filled. For example, for the OR datasets, the minimum item size is 20 and the best fit heuristic may leave gaps of size 18 and 19 which will never be filled. In contrast, *FunSearch* tends to avoid such gaps (see Figure E.25 for a visualization of an example).

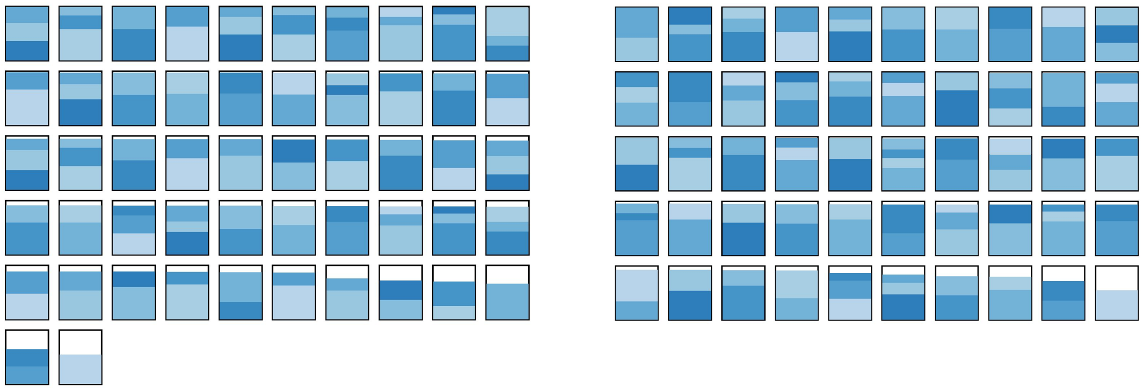

**Figure E.25:** Packing solutions on u120_07 from the OR1 bin packing dataset sorted by remaining capacity. *Left*: Best fit. *Right*: *FunSearch* (the heuristic in Figure C.13). As can be seen, best fit leaves several gaps that are slightly smaller than the smallest items, leading to worse performance.

# F Implementation of *FunSearch*

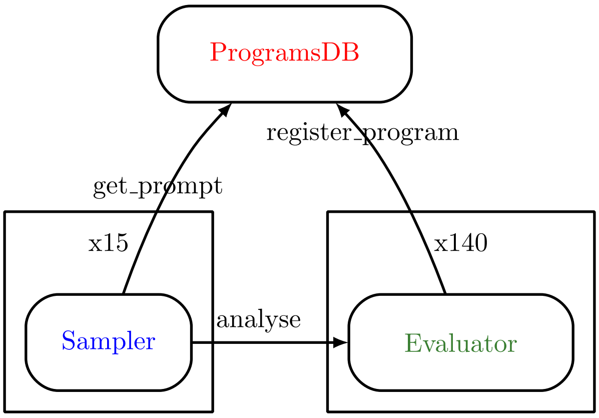


**Figure F.26:** *FunSearch* graph.

In this section we present a pseudocode implementation of *FunSearch*. The function in Algorithm F.1 constructs the graph in Figure F.26 and starts the whole procedure.

**Algorithm F.1** Function that launches a *FunSearch* experiment.

```
def main (specification, tests_inputs, num_samplers = 15, num_evaluators = 140)
  function_to_evolve, function_to_run = extract_function_names (specification)
  # We create a singleton ProgramsDB.
  programs_db = ProgramsDB (function_to_evolve)
  # We create a set of evaluators.
  evaluators = []
  for i ∈ {1, . . . , num_evaluators} do
      evaluators. append (Evaluator (programs_db, function_to_run, tests_inputs))
  # And we create a set of samplers.
  samplers = []
  for i ∈ {1, . . . , num_samplers} do
      samplers. append (Sampler (programs_db, evaluators))
  # We send the initial implementation in the specification for analysis by one of the evaluators.
  evaluators[0] . analyse (specification)
  # Finally we start calling the sample function of all samplers in parallel.
  for sampler ∈ samplers do
      sampler. sample ()
```

This function uses 3 classes of objects: Sampler, Evaluator and ProgramDB. For each of those, we present its pseudocode implementation in Appendix F.1, Appendix F.2 and Appendix F.3 respectively.

## F.1 Sampler

Class for sampling using an LLM.

**Algorithm F.2** Constructor of the Sampler class.

```
def constructor (self, programs_db, evaluators, samples_per_prompt = 4)
  self. programs_db = programs_db
  self. evaluators = evaluators
  self. llm = LLM (samples_per_prompt)
```

**Algorithm F.3** Getting prompts and sampling in an endless loop.

```
def sample (self)
  while True do
      prompt, island_id = self. programs_db. get_prompt ()
      samples = self. llm. draw_samples (prompt)
      for sample ∈ samples do
          pick_at_random (self. evaluators) . analyse (sample, island_id)
```

## F.2 Evaluator

Class that analyses functions generated by LLMs and send the results to the Programs Database. The Evaluator has access to a sandbox to run programs. The sandbox is needed for two reasons. The first reason is to prevent the given program doing things we do not want it to do, like connecting to the internet or using too much memory. The second is to stop the program after a given timeout, so that we do not need to wait for a program that takes too long, or even infinity. The sandbox returns two outputs. The first one is the actual output obtained when running the given program, and the second one is a boolean that indicates whether the program runs correctly, i.e. if it did not contain syntactic or runtime errors. If the program executed correctly, its actual output is assumed to be the numerical score of the corresponding solution, or $\emptyset$ if the solution was invalid (cf the `main` functions in Figure 2).

**Algorithm F.4** Constructor of the Evaluator class.

```
def constructor (self, programs_db, function_to_run, test_inputs, timeout_seconds = 30)
  self. programs_db = programs_db
  self. function_to_run = function_to_run
  self. test_inputs = test_inputs
  self. T = timeout_seconds
```

**Algorithm F.5** Analyses a given program.

```
def analyse (self, program, island_id)
  scores_per_test = {}
  for test_input ∈ self. test_inputs do
      test_output, runs_ok = sandbox_run (program, self. function_to_run, test_input, self. T)
      if runs_ok and not calls_ancestor (program) and test_output ≠ ∅ then
          scores_per_test[test_input] = test_output
  # If at least it passed one test, we send it to the ProgramsDB.
  if scores_per_test ≠ {} then
      self. programs_db. register_program (program, island_id, scores_per_test)
```

## F.3 ProgramsDB

Class that stores and serves the programs. It uses a set of instances from the class Island whose pseudocode is shown in Appendix F.3.1. In turn, that class relies on using instances of the class Cluster, whose pseudocode is in Appendix F.3.2.

**Algorithm F.6** Constructor of the ProgramsDB class.

```
def constructor(self, function_to_evolve, number_islands = 10, reset_period = 4 * 60 * 60, functions_per_prompt = 2, temperature = 0.1, temperature_period = 30000)
    self.k = functions_per_prompt
    self.T_0 = temperature
    self.N = temperature_period
    self.function_to_evolve = function_to_evolve
    self.islands = []
    for i ∈ {1, ..., number_islands} do
        self.islands.append(Island(self.function_to_evolve, self.k, self.T_0, self.N))
    self.islands_to_reset = number_islands / 2
    self.best_score_per_island = [−∞] * number_islands
    self.best_program_per_island = [∅] * number_islands
    self.best_scores_per_test_per_island = [∅] * number_islands
    self.reset_period = reset_period
    self.last_reset_time = time_now()
```

**Algorithm F.7** Returns a new prompt.

```
def get_prompt(self)
    island_id = pick_at_random({0, ..., |self.islands| − 1})
    return self.islands[island_id].get_prompt(), island_id
```

**Algorithm F.8** Registers the given program into the given island.

```
def register_program_in_island(self, program, island_id, scores_per_test)
    self.islands[island_id].register_program(program, scores_per_test)
    score = reduce_score(scores_per_test)
    if score > self.best_score_per_island[island_id] then
        self.best_program_per_island[island_id] = program
        self.best_score_per_island[island_id] = score
        self.best_scores_per_test_per_island[island_id] = scores_per_test
```

**Algorithm F.9** Registers the given program into the database.

```
def register_program (self, program, island_id, scores_per_test)
if island_id = ∅ then
# This is a program added at the beginning, so adding to all islands
    for island_id ∈ {0, . . . , | self. islands | − 1} do
        self. register_program_in_island (program, island_id, scores_per_test)
else
    self. register_program_in_island (program, island_id, scores_per_test)
# Check whether it is time to reset some islands
if time_now () − self. last_reset_time > self. reset_period then
    last_reset_time = time_now ()
    self. reset_islands ()
```

**Algorithm F.10** Restarts the population of a proportion of the islands.

```
def reset_islands (self)
# We sort best scores after adding minor noise to break ties.
sorted_islands_scores = argsort (add_gaussian_noise (self. best_score_per_island, 10^−6))
reset_islands_ids = []
keep_islands_ids = []
for i ∈ 0, . . . , | sorted_islands_scores | − 1 do
    if i < self. islands_to_reset then
        reset_islands_ids. apppend (sorted_islands_scores[i])
    else
        keep_islands_ids. append (sorted_islands_scores[i])
for island_id ∈ reset_islands_ids do
    self. islands[island_id] = Island (self. function_to_evolve, self. k, self. T_0, self. N)
    self. best_score_per_island[island_id] = −∞
    founder_island_id = pick_at_random (keep_islands_ids)
    founder = self. best_program_per_island[founder_island_id]
    founder_scores = self. best_scores_per_test_per_island[founder_island_id]
    self. register_program_in_island (founder, island_id, founder_scores)
```

### F.3.1 Island

**Algorithm F.11** Constructor of the Island class.

**def** constructor (self, function_to_evolve, functions_per_prompt, temperature, temperature_period)

self. function_to_evolve = function_to_evolve
self. functions_per_prompt = functions_per_prompt
self. clusters = {}
self. $T_0$ = temperature
self. $N$ = temperature_period
self. template = $\emptyset$
*# Total number of programs registered on this island.*
self. $n = 0$

**Algorithm F.12** Adds the given program into the island.

**def** register_program (self, program, scores_per_test)

**if** not self. template **then**
    self. template = extract_template_from_program (program)
**if** scores_per_test $\notin$ self. clusters **then**
    score = reduce_score (scores_per_test)
    self. clusters[scores_per_test] = Cluster (score, program)
**else**
    self. clusters[scores_per_test]. programs. append (program)
self. $n$ = self. $n + 1$

**Algorithm F.13** Builds and returns a prompt.

**def** get_prompt (self)

$s = []$

**for** cluster $\in$ self. clusters **do**

    $s$ . append (cluster. score)

$T_{\text{cluster}} = \text{self.}\, T_0 \cdot \left(1 - \frac{\text{self.}\, n \bmod \text{self.}\, N}{\text{self.}\, N}\right)$

$p_i = \frac{\exp(s_i / T_{\text{cluster}})}{\sum_{i'} \exp(s_{i'} / T_{\text{cluster}})}$

*# At the beginning we might not have enough clusters.*

functions_per_prompt = min (| self. clusters |, self. functions_per_prompt)

chosen_clusters = pick_at_random (self. clusters, $p$, functions_per_prompt)

programs = []

scores = []

**for** cluster $\in$ chosen_clusters **do**

    programs. append (cluster. sample_program ())

    scores. append (cluster. score)

sorted_programs = []

indices = argsort (scores)

**for** index $\in$ indices **do**

    sorted_programs. append (programs[index])

**return** self. generate_prompt (sorted_programs)

**Algorithm F.14** Creates a prompt containing a sequence of function implementations.

**def** generate_prompt (self, implementations)

prompt = self. template

**for** $i \in \{0, \ldots, |\,\text{implementations}\,| - 1\}$ **do**

    new_function_name = get_versioned_function_name (self. function_to_evolve, $i$)

*# Rename the function, including any internal recursive calls, and update its docstring.*

    new_implementation = rename_function (implementations[$i$], new_function_name)

    prompt = string_concatenation ([prompt, new_implementation], double_line)

*# Create the header of the function to be generated by the LLM.*

version_generated = | implementations |

new_function_name = get_versioned_function_name (self. function_to_evolve, version_generated)

header = make_header_like (implementations[0], new_function_name)

*# Rename the call to the target function.*

prompt = rename_function_calls (prompt, self. function_to_evolve, new_function_name)

prompt = string_concatenation ([prompt, implementation], double_line)

**return** prompt

#### F.3.2 Cluster

**Algorithm F.15** Constructor of a Cluster.

**def** constructor (self, score, first_program)
1: self. score = score
2: self. programs = [first_program]

**Algorithm F.16** Samples a program giving higher probability to shorter programs.

**def** sample_program (self, $T_{\text{program}} = 1$)
1: $\ell = []$
2: **for** program ∈ self. programs **do**
3: $\quad \ell$ . append (− length (program))
4: $\widetilde{\ell}_i = \frac{\ell_i - \min_{i'} \ell_{i'}}{\max_{i'} \ell_{i'} + 10^{-6}}$
5: $p_i = \frac{\exp\left(\widetilde{\ell}_i / T_{\text{program}}\right)}{\sum_{i'} \exp\left(\widetilde{\ell}_{i'} / T_{\text{program}}\right)}$
6: **return** pick_at_random (self. programs, $p$)

# References


[1] Code models overview. https://cloud.google.com/vertex-ai/docs/generative-ai/code/code-models-overview, 2023. [Online; accessed July-2023].

[2] Raymond Li, Loubna Ben Allal, Yangtian Zi, Niklas Muennighoff, Denis Kocetkov, Chenghao Mou, Marc Marone, Christopher Akiki, Jia Li, Jenny Chim, et al. StarCoder: may the source be with you! *arXiv preprint arXiv:2305.06161*, 2023.

[3] X. Chen, C. Liang, D. Huang, E. Real, K. Wang, Y. Liu, H. Pham, X. Dong, T. Luong, C.-J. Hsieh, Y. Lu, and Q. V. Le. Symbolic discovery of optimization algorithms. *arXiv preprint arXiv:2302.06675*, 2023.

[4] Charles R. Harris, K. Jarrod Millman, Stéfan J. van der Walt, Ralf Gommers, Pauli Virtanen, David Cournapeau, Eric Wieser, Julian Taylor, Sebastian Berg, Nathaniel J. Smith, Robert Kern, Matti Picus, Stephan Hoyer, Marten H. van Kerkwijk, Matthew Brett, Allan Haldane, Jaime Fernández del Río, Mark Wiebe, Pearu Peterson, Pierre Gérard-Marchant, Kevin Sheppard, Tyler Reddy, Warren Weckesser, Hameer Abbasi, Christoph Gohlke, and Travis E. Oliphant. Array programming with NumPy. *Nature*, 585(7825):357–362, September 2020.

[5] Guido Van Rossum and Fred L. Drake. *Python 3 Reference Manual*. CreateSpace, Scotts Valley, CA, 2009.

[6] Fred Tyrrell. New lower bounds for cap sets. *arXiv preprint arXiv:2209.10045*, 2022.

[7] Laurent Perron and Frédéric Didier. Cp-sat.

[8] C. E. Shannon. The zero error capacity of a noisy channel. *IRE Transactions on Information Theory*, IT-2(3):8–16, 1956.

[9] J. Körner and A. Orlitsky. Zero-error information theory. *IEEE Transactions on Information Theory*, (44):2207–2229, 1998.

[10] N. Alon. The Shannon capacity of a union. *Combinatorica*, (18):301–310, 1998.

[11] J. Zuiddam. The asymptotic spectrum of graphs and the Shannon capacity. *Combinatorica*, (39):1173–1184, 2019.

[12] T. Bohman. A limit theorem for the Shannon capacities of odd cycles I. *Proceedings of the American Mathematical Society*, (131):3559–3569, 2003.

[13] C. D. Godsil. Problems in algebraic combinatorics. *Electronic Journal of Combinatorics*, (2):1–20, 1995.

[14] L. Lovász. On the Shannon capacity of a graph. *IEEE Transactions on Information Theory*, IT-25(1):1–7, 1979.

[15] W. H. Haemers. An upper bound for the Shannon capacity of a graph. *Colloquia Mathematica Societatis János Bolyai*, 25:267–272, 1978.

[16] L. Baumert. A combinatorial packing problem. In *SIAM-AMS Proceedings*, number 131, pages 97–108, 1971.

[17] A. Vesel and J. Zerovnik. Improved lower bound on the Shannon capacity of $C_7$. *Information Processing Letters*, 81(5):277–282, 2002.

[18] F. J. R. Ruiz and F. Perez-Cruz. Zero-error codes for the noisy-typewriter channel. In *IEEE Information Theory Workshop*, pages 495–497, 2011.

[19] K. A. Mathew and R. J. Östergård. New lower bounds for the Shannon capacity of odd cycles. *Designs, Codes and Cryptography*, (84):13–22, 2016.

[20] S. C. Polak and A. Schrijver. New lower bound on the Shannon capacity of $C_7$ from circular graphs. *Information Processing Letters*, 143:37–40, 2019.

[21] Ben Green. Finite field models in additive combinatorics. *arXiv preprint math/0409420*, 2004.

[22] A. K. Chandra, M. L. Furst, and R. J. Lipton. Multi-party protocols. In *Proceedings of the 15th Annual ACM Symposium on Theory of Computing*, pages 94–99, 1983.

[23] A. Shraibman. A note on multiparty communication complexity and the Hales-Jewett theorem. *Information Processing Letters*, 139:44–48, 2018.

[24] N. Linial, T. Pitassi, and A. Shraibman. On the communication complexity of high-dimensional permutations. In *Innovations in Theoretical Computer Science Conference*, volume 124 of *Leibniz International Proceedings in Informatics*, pages 54:1–54:20, 2018.

[25] N. Alon and A. Shraibman. Algorithmic number on the forehead protocols yielding dense Ruzsa-Szemerédi graphs and hypergraphs. *arXiv preprint arXiv:2001.00387*, 2020.

[26] N. Linial and A. Shraibman. Larger corner-free sets from better NOF exactly-n protocols. *arXiv preprint arXiv:2102.00421*, 2021.

[27] M. Christandl, O. Fawzi, H. Ta, and J. Zuiddam. Larger corner-free sets from combinatorial degenerations. *arXiv preprint arXiv:2111.08262*, 2021.

[28] Ben Green. Lower bounds for corner-free sets. *arXiv preprint arXiv:2102.11702*, 2021.

[29] Yves Edel. Extensions of generalized product caps. *Designs, Codes and Cryptography*, 31:5–14, 2004.

[30] Raymond Hill. On the largest size of cap in $S_{5,3}$. *Atti della Accademia Nazionale dei Lincei. Classe di Scienze Fisiche, Matematiche e Naturali. Rendiconti*, 54(3):378–384, 1973.

[31] Peter Jephson Cameron and Jacobus Hendricus Van Lint. *Designs, graphs, codes and their links*, volume 3. Cambridge University Press Cambridge, 1991.

[32] John E Beasley. Or-library: distributing test problems by electronic mail. *Journal of the operational research society*, 41(11):1069–1072, 1990.

[33] Spyros Angelopoulos, Shahin Kamali, and Kimia Shadkami. Online bin packing with predictions. *arXiv preprint arXiv:2102.03311*, 2021.
